# ESC‐sEVs Rejuvenate Senescent Hippocampal NSCs by Activating Lysosomes to Improve Cognitive Dysfunction in Vascular Dementia

**DOI:** 10.1002/advs.201903330

**Published:** 2020-03-20

**Authors:** Guowen Hu, Yuguo Xia, Juntao Zhang, Yu Chen, Ji Yuan, Xin Niu, Bizeng Zhao, Qing Li, Yang Wang, Zhifeng Deng

**Affiliations:** ^1^ Department of Neurosurgery Shanghai Jiaotong University Affiliated Sixth People's Hospital Shanghai 200233 China; ^2^ Institute of Microsurgery on Extremities Shanghai Jiaotong University Affiliated Sixth People's Hospital Shanghai 200233 China; ^3^ Department of Orthopedic Surgery Shanghai Jiao Tong University Affiliated Sixth People's Hospital Shanghai 200233 China

**Keywords:** embryonic stem cells derived small extracellular vesicles (ESC‐sEVs), hippocampal neural stem cells (HNSCs), lysosomes, senescence, vascular dementia

## Abstract

Vascular dementia (VD) is one of the most common types of dementia, however, the intrinsic mechanism is unclear and there is still lack of effective medications. In this study, the VD rats exhibit a progressive cognitive impairment, as well as a time‐related increasing in hippocampal neural stem cells (H‐NSCs) senescence, lost and neurogenesis decline. Then, embryonic stem cell‐derived small extracellular vesicles (ESC‐sEVs) are intravenously injected into VD rats. ESC‐sEVs treatment significantly alleviates H‐NSCs senescence, recovers compromised proliferation and neuron differentiation capacity, and reverses cognitive impairment. By microarray analysis and RT‐qPCR it is identified that several miRNAs including miR‐17‐5p, miR‐18a‐5p, miR‐21‐5p, miR‐29a‐3p, and let‐7a‐5p, that can inhibit mTORC1 activation, exist in ESC‐sEVs. ESC‐sEVs rejuvenate H‐NSCs senescence partly by transferring these miRNAs to inhibit mTORC1 activation, promote transcription factor EB (TFEB) nuclear translocation and lysosome resumption. Taken together, these data indicate that H‐NSCs senescence cause cell depletion, neurogenesis reduction, and cognitive impairment in VD. ESC‐sEVs treatment ameliorates H‐NSCs senescence by inhibiting mTORC1 activation, and promoting TFEB nuclear translocation and lysosome resumption, thereby reversing senescence‐related neurogenesis dysfunction and cognitive impairment in VD. The application of ESC‐sEVs may be a novel cell‐free therapeutic tool for patients with VD, as well as other aging‐related diseases.

## Graphical abstract

Hippocampal neural stem cells (H‐NSCs) senescence causes their loss and neurogenesis decline, resulting in cognitive impairment in vascular dementia (VD). Embryonic stem cells derived small extracellular vesicles (ESC‐sEVs) can rejuvenate H‐NSCs senescence by transferring miRNAs including miR‐17‐5p, miR‐18a‐5p, miR‐21‐5p, miR‐29a‐3p, and let‐7a‐5p to inhibit mTORC1 activation, and promote transcription factor EB nuclear translocation and lysosome resumption, which reverses senescence‐related neurogenesis dysfunction and cognitive impairment in VD.

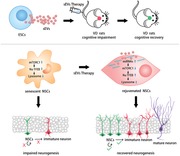

## 1 Introduction

Vascular dementia (VD) is a severe cognitive impairment syndrome, characterized by a progressive memory and behavioral deterioration, accounting for roughly 17–20% of all dementia patients and only preceded by Alzheimer's disease (AD).^[^ ^1^ ^]^ Chronic cerebral hypoperfusion (CCH) in VD induced ischemia, oxidative stress, and neuroinflammation damaged neuron and declined synaptic plasticity in hippocampus, were considered the main pathophysiological process of VD.^[^ ^2^, ^3^ ^]^ Hippocampus is the key structure involved in the formation of cognition and is extremely sensitive to cerebral hypoperfusion. Hippocampal neural stem cells (H‐NSCs) located in the subgranular zone (SGZ) of the dentate gyrus (DG). H‐NSCs and their neurogenesis play crucial roles in keeping and restoring of hippocampus‐dependent brain functions.^[^ ^4^ ^]^ In aged brain or neurodegenerative disorders like AD, the number of H‐NSCs and their neurogenesis declined, leading to neuroplasticity reduction and cognitive dysfunction.^[^ ^5^, ^6^ ^]^ However, the physiological change of H‐NSCs in VD is still unclear.

Cellular senescence, which is an essentially irreversible cell cycle arrest caused by various biological and pathological conditions, has been demonstrated to account for tissue homeostasis impairment.^[^ ^7^ ^]^ Stem cells senescence has been confirmed as an important pathogenesis for multiple disorders.^[^ ^8^ ^]^ For example, senescent hematopoietic stem cells lose their self‐renewal and regenerative potential, and weaken the adaptive immune system because the cells are more likely to differentiate toward the myeloid lineage at the expense of the lymphoid lineage.^[^ ^9^ ^]^ Senescent mesenchymal stem cells are responsible for bone‐related disease because their decreased propensity for proliferation and osteogenic differentiation, as well as increased expression of pro‐inflammatory factors.^[^ ^10^ ^]^ Multiple studies have demonstrated that H‐NSCs senescence was associated with stem cell markers lose,^[^ ^11^ ^]^ as well as self‐renewal and neurogenic capacity impairment,^[^ ^12^, ^13^, ^14^ ^]^ indicating that NSCs senescence may contribute to their decline in neurogenic niches and brain dysfunction.^[^ ^15^, ^16^ ^]^ CCH induced abnormal O_2_ levels, neuroinflammation, and oxidative stresses are crucial factors that may lead to cell senescence.^[^ ^17^ ^]^ Thus, we hypothesized that during the progress of VD, H‐NSCs may get senescence, which resulted in H‐NSCs reduction and neurogenesis decline, and finally proceed to cognitive dysfunction.

Rejuvenation of stem cells senescence can enhance their self‐renewal and regenerative capacity, which has been proposed as a promising therapeutic approach in restoring tissue structure and function.^[^ ^8^ ^]^ Small extracellular vesicles (sEVs), including classical exosomes, are natural nanosized particles that participate in intracellular communication and influence recipient cells behavior via the delivery of functional biomolecules.^[^ ^18^ ^]^ Stem cells derived sEVs are regarded as an attractive therapeutic strategy in regenerative medicine, for their promising pro‐regenerative effects, devoid of risk of aneuploidy and low possibility of immune rejection.^[^ ^19^, ^20^, ^21^ ^]^ Embryonic stem cells derived sEVs (ESC‐sEVs) contain bioactive factors from their parental cells which possess unique capacities including infinite proliferation ability, pluripotency, and intrinsic youthful barrier to aging, suggesting they may be beneficial to aging‐associated disease.^[^ ^22^ ^]^ Recent studies have demonstrated that ESC‐sEVs can rejuvenate somatic cell senescence and promote regeneration.^[^ ^23^, ^24^ ^]^ However, whether ESC‐sEVs could ameliorate NSCs senescence has not been reported.

In this study, we reported that in the progress of VD, the senescence of H‐NSCs lead to neurogenesis decline and cognitive impairment. ESC‐sEVs can recovery cognitive dysfunction in VD partly by transferring miRNAs to inhibit mTORC1 activation, promote TFEB nuclear translocation and lysosome resumption, which finally resulted in senescent H‐NSCs rejuvenation. Here, we show for the first time that H‐NSCs senescence contribute to cognitive impairment in VD, and ESC‐sEVs have the potential to reverse cognitive dysfunction by rejuvenating H‐NSCs senescence.

## 2 Results

### 2.1 Hippocampal NSCs Exhibited Senescent Phenotype in VD Rats

Progressive cognitive and behavioral deterioration is the key characteristic of VD, thus, we first detected the spatial learning and memory abilities by MWM.^[^ ^25^ ^]^ As shown in Figure S1a (Supporting Information), compared with sham group at 0.5, 1, 2, 4, and 8 months post‐surgery respectively, the escape latency on day 4 in VD group was much longer. Besides, the escape latency on day 4 in VD group increased gradually from 0.5 to 8 months. The time spent in the target quadrant on day 5 was also markedly longer in sham group at each time point. As synaptic plasticity is the neurobiological basis of cognitive function,^[^ ^26^ ^]^ we then detected the expression of hippocampal synapse‐related proteins, including synaptophysin (Syp), postsynaptic density protein‐95 (Psd‐95), growth associated protein (Gap‐43), and synapsin IIa (Syn‐IIa) in VD and sham group. As shown in Figure S1b (Supporting Information), the expression levels of Syp, Psd‐95, Gap43, and Syn‐IIa in VD group gradually decreased from 0.5 to 8 months, and were much lower than those in sham group at 1, 2, 4, and 8 months respectively. These data confirmed a time‐related reduction of cognitive functions in VD.

**Figure 1:**
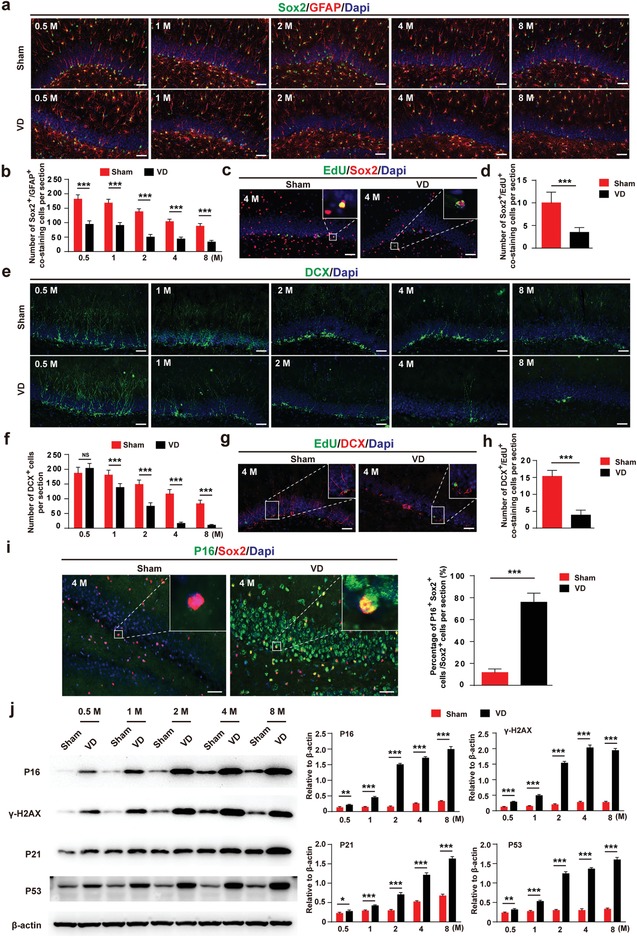
H‐NSCs and neurogenesis decline, and H‐NSCs senescence in the SGZ of VD rats. a) Immunofluorescence staining of Sox2^+^ (green) and GFAP^+^ (red) in hippocampus and their b) estimated number at each time point, as well as c) EdU^+^ (green) and Sox2^+^ (red) in hippocampus and their d) estimated number at 4 months, in sham and VD group. (Scale bar, 50 µm; *n* = 6/group; ***P < 0.001). e) Immunofluorescence staining of hippocampal immature neuron ( DCX^+^, green) and their f) estimated number at each time point, as well as g) proliferative immature neuron (EdU^+^, green; DCX^+^, red) and their h) estimated number at 4 months, in sham and VD group. (Scale bar, 50 µm; *n* = 6/group; ***P < 0.001). i) Immunofluorescence staining of senescent H‐NSCs (p16^INK4a+^, green; Sox2^+^, red) and their estimated number in sham and VD group at 4 months. (Scale bar, 50 µm; *n* = 6/group; ***P < 0.001). j) Western blot analysis and quantification of p16^INK4a^, γ ‐H2AX, P21, and P53 in isolated neurospheres in sham and VD group at each time point (*n* = 3/group; *P < 0.05, **P < 0.01, ***P < 0.001).

H‐NSCs play crucial role in keeping and restoring of cognition, their decline directly lead to the reduction of neuroplasticity and cognitive function.^[^ ^5^, ^27^ ^]^ Therefore, we calculated the number of H‐NSCs (Sox2^+^/GFAP^+^) and newly generated immature neurons (DCX^+^),^[^ ^28^ ^]^ as well as their proliferation status (EdU^+^) in SGZ from 0.5 to 8 months. Interestingly, compared to sham group, the number of Sox2^+^/GFAP^+^ cells was much lower in VD group at each time point (**Figure** **1**a,b). The number of Sox2^+^ cells incorporating EdU was also decreased in VD group at 4 months (Figure 1c,d). Besides, the number of DCX^+^ cells in VD group was much lesser than that of sham group since 1 month post‐surgery (Figure 1e,f). The number of DCX^+^/EdU^+^ cells in VD group was also reduced at 4 months (Figure 1g,h). These observations showed a clear reduction of H‐NSCs and neurogenesis in VD, which may be responsible for the progressive cognitive dysfunction. Furthermore, we isolated H‐NSCs from hippocampus of VD rats to detect their proliferation and differentiation ability in vitro. As shown in Figure S2a (Supporting Information), the percentage of proliferating H‐NSCs (EdU^+^/Nestin^+^/Dapi^+^cells) was much lower in VD group at 1, 2, 4, and 8 months respectively. After induction, the percentage of differentiated neurons (β‐III tubulin^+^ cells) in VD group was also significantly reduced at each time point from 0.5 to 8 months (Figure S2b, Supporting Information). These results indicated that the proliferation and neuronal differentiation capacity of H‐NSCs in VD group are decreased, which may responsible for H‐NSCs and neurogenesis reduction.

Next, we detected the senescent status of H‐NSCs. As shown in Figure S3a (Supporting Information), compared to sham group, the activity of senescence‐associated β‐galactosidase (SA‐β‐gal) significantly increased in hippocampus of VD group at each time point from 0.5 to 8 months, suggesting an aggravation of hippocampal senescence in VD rats. Then we performed p16^INK4a^ and Sox2 double staining to identify senescent NSCs in hippocampal slices of VD in 4 months. As shown in Figure 1i, the percentage of Sox2^+^/p16^INK4a^ cells in total Sox2^+^ cells in SGZ was much higher in VD group. Additionally, SA‐β‐gal staining of isolated hippocampal neurospheres showed much higher SA‐β‐gal activity in VD group at 0.5, 1, 2, 4, and 8 months respectively (Figure S3b, Supporting Information). Moreover, the expression of senescence‐related proteins (p16^INK4a^, γ‐H2AX, P21, P53) in isolated hippocampal neurospheres gradually increased in VD group with time after operation (Figure 1j). These findings indicated a time‐related H‐NSCs senescence in VD rats. Taken together, these results suggest that during the progress of VD, the senescence of H‐NSCs resulted in their loss, neurogenesis reduction, and subsequently cognitive dysfunction.

### 2.2 ESC‐sEVs Ameliorate Hippocampal NSCs Senescence and Enhanced NSCs Activity in VD

**Figure 2:**
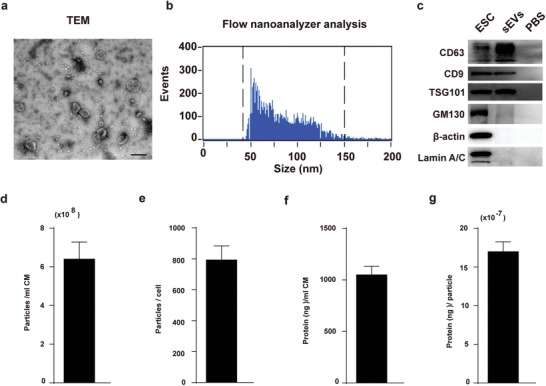
Characterization of ESC‐sEVs. a) Morphology of ESC‐sEVs observed by TEM (Scale bar, 200 nm). b) Particle size distribution and concentration of ESC‐sEVs measured by nanoflow cytometer. c) Western blot showed the presence of sEVs markers including CD63, CD9, and TSG101, and negative for GM130, β‐actin, and Lamin A/C. The yield of ESC‐sEVs was evaluated in terms of particle concentration and protein concentration, the mean particle concentration was d) 6.41 × 10^8^ ± 0.89 × 10^8^ particles per mL CM and e) 792.83 ± 91.08 particles per cell, f) the mean protein concentration was 1049.82 ± 84.14 ng per mL CM and g) 16.98 × 10^−7^ ± 1.27 × 10^−7^ ng per particle (*n* = 6).

As described above, H‐NSCs senescence is an important reason for their loss and neurogenesis reduction in VD. Thus, amelioration of H‐NSCs senescence may reverse hippocampal neurogenesis reduction and cognitive impairment in VD. In our previous study, we demonstrated that ESC‐sEVs possess excellent ability in rejuvenating endothelial senescence.^[^ ^24^ ^]^ Therefore, we explored whether ESC‐sEVs could rejuvenate H‐NSCs senescence in VD. ESCs colonies were identified with the expression of alkaline phosphatase (ALP) and pluripotency‐related markers including OCT4, Nanog, TRA‐1‐81, TRA‐1‐60, and SSEA4 (Figure S4a–c, Supporting Information). Then, ESC‐sEVs were purified from the conditional medium (CM). ESC‐sEVs exhibited a size distribution mostly around 100 nm and with a characteristic cup‐shaped morphology under transmission electron microscope (TEM) (**Figure** **2**a). Flow nanoanalyzer analysis indicated particles with a mean diameter of 72.4 ± 21.3 nm and a concentration of 1.93 × 10^11^ ± 0.16 × 10^11^ particles per mL (Figure 2b). Western blot showed ESC‐sEVs express exosomal markers CD9, CD63, and TSG101, but not the Golgi matrix protein GM130, β‐actin, and Lamin A/C (Figure 2c), which means no contamination of cellular components in isolated ESC‐sEVs. We further evaluated the yield of ESC‐sEVs by particle concentration and protein concentration. As shown in Figure 2d–g, the mean particle concentration was 6.41 × 10^8^ ± 0.89 × 10^8^ particles per mL CM (Figure 2d) and 792.83 ± 91.08 particles per cell (Figure 2e). The mean protein concentration was 1049.82 ± 84.14 ng per mL CM (Figure 2f) and 16.98 × 10^−7^ ± 1.27 × 10^−7^ ng per particle (Figure 2g). We have submitted all relevant data of our experiments to the EV‐TRACK knowledgebase (EV‐TRACK ID: EV190089).^[^ ^29^ ^]^ The EV‐metric score is 56%.

**Figure 3:**
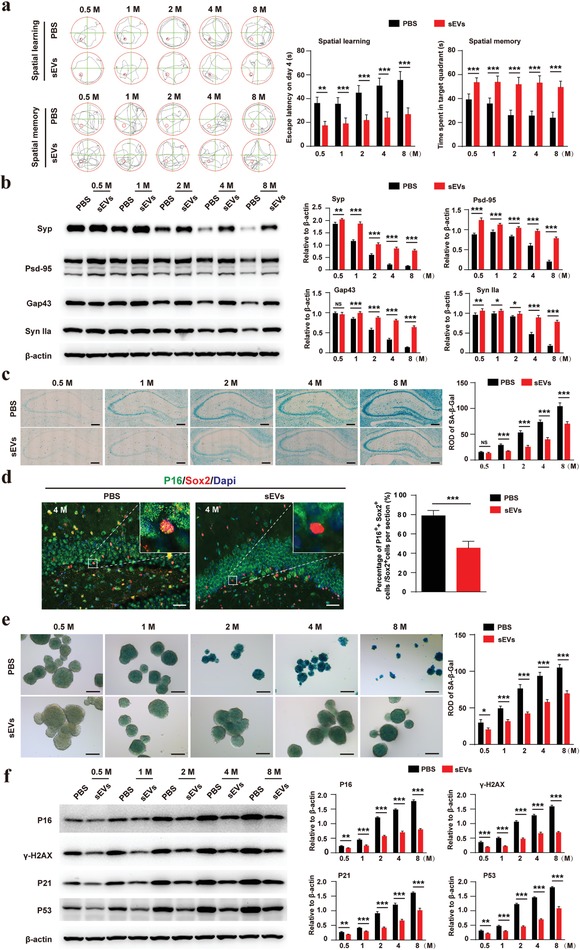
ESC‐sEVs reverse cognitive impairment and rejuvenate H‐NSCs senescence. a) Spatial learning and memory abilities in ESC‐sEVs and PBS group were tested by MWM (*n* = 10/group; **P < 0.01, ***P < 0.001). b) Western blot analysis and quantitative of Syp, GAP43, PSD95, and Syn IIa in hippocampus of ESC‐sEVs and PBS group at each time point (*n* = 6/group; *P < 0.05, **P < 0.01, ***P < 0.001). c) SA‐β‐gal staining of hippocampus and quantification of SA‐β‐gal intensity in ESC‐sEVs and PBS group at each time point (Scale bar, 400 µm; *n* = 6/group; ***P < 0.001). d) Immunofluorescence staining of senescent H‐NSCs (p16^INK4a+^, green; Sox2^+^, red) and their estimated number in ESC‐sEVs and PBS group at 4 months (Scale bar, 50 µm; *n* = 6/group; ***P < 0.001). e) SA‐β‐gal staining of isolated neurospheres and quantification of SA‐β‐gal intensity in ESC‐sEVs and PBS group at each time point (Scale bar, 100 µm; *n* = 3/group; *P < 0.05, ***P < 0.001). f) Western blot analysis and quantification of p16^INK4a^, γ‐H2AX, P21, and P53 in isolated neurospheres in ESC‐sEVs and PBS group at each time point (*n* = 3/group; **P < 0.01, ***P < 0.001).

Next, ESC‐sEVs were injected intravenously to the VD rats. As shown in Figure S5a (Supporting Information), DiR‐labeled ESC‐sEVs treated rats exhibited visibly high levels of fluorescence intensity in the brain compared to PBS treated rats at 6 h after administration, confirming the ability of ESC‐sEVs to migrate into the brain of VD rat. We then investigated the therapeutic effects of ESC‐sEVs in VD rats. As shown in **Figure** **3**a, the escape latency was shorter while the duration in target quadrant was much longer in ESC‐sEVs group than that of the PBS group at each time point from 0.5 to 8 months. Moreover, the decreased expression of hippocampal Syp, Psd‐95, Gap‐43, and Syn‐IIa in VD rats were rescued by ESC‐sEVs treatment (Figure 3b). These data indicated that the application of ESC‐sEVs could reverse cognitive deficit in VD. To investigate whether reversion of cognitive dysfunction in VD by ESC‐sEVs was through ameliorating H‐NSCs senescence, we tested senescence associated hallmarks in hippocampus. As shown in Figure 3c, hippocampal SA‐β‐gal activity in ESC‐sEVs group was reduced at each time point from 1 to 8 months. The percentage of Sox2^+^/p16^INK4a+^ cells in total Sox2^+^ cells in SGZ also decreased in ESC‐sEVs group at 4 months post‐surgery (Figure 3d). Subsequently, H‐NSCs were isolated from hippocampus and tested for senescent phenotypes. SA‐β‐gal activity in ESC‐sEVs group was significantly decreased at each time point from 0.5 to 8 months (Figure 3e). The expression of p16^INK4a^, γ ‐H2AX, P21, and P53 in ESC‐sEVs group were also reduced compared to that of PBS group at 0.5, 1, 2, 4, and 8 months respectively (Figure 3f). These results indicated that ESC‐sEVs treatment could decelerate H‐NSCs senescence in VD.

**Figure 4:**
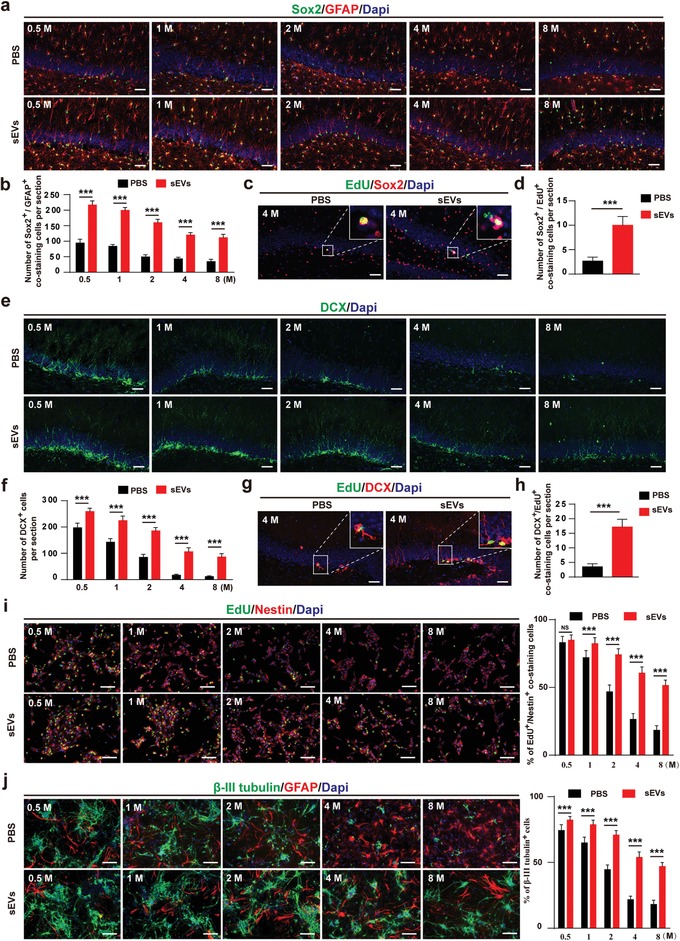
ESC‐sEVs reverse H‐NSCs and neurogenesis decline in the SGZ of VD rats. a) Immunofluorescence staining of H‐NSCs (Sox2^+^, green; GFAP^+^, red) and their b) estimated number at each time point, c) as well as proliferated H‐NSCs ( EdU^+^, green; Sox2^+^, red) and their d) estimated number at 4 months, in ESC‐sEVs and PBS group (Scale bar, 50 µm; *n* = 6/group; ***P < 0.001). e) Immunofluorescence staining of hippocampal immature neuron (DCX^+^, green) and their f) estimated number at each time point, g) as well as proliferated immature neuron (EdU^+^, green; DCX^+^, red) and their h) estimated number at 4 months, in ESC‐sEVs and PBS group (Scale bar, 50 µm; *n* = 6/group; ***P < 0.001). i) Immunofluorescence staining of EdU incorporation and quantification of EdU^+^/Nestin^+^/Dapi^+^ cells in Nestin^+^/Dapi^+^ cells in ESC‐sEVs and PBS group at each time point (Scale bar, 100 µm; *n* = 3/group; ***P < 0.001). j) Immunofluorescence staining of neuron differentiation and quantification of β‐III tubulin^+^ cells in whole cells in ESC‐sEVs and PBS group at each time point (Scale bar, 100 µm; *n* = 3/group; ***P < 0.001).

Furthermore, we detected the number of NSCs and immature neuron in SGZ. As shown in **Figure** **4**a,b,e,f, the number of Sox2^+^/GFAP^+^ cells and DCX^+^ cells were much higher in ESC‐sEVs group at indicated time after surgery. The number of proliferating NSCs (Figure 4c,d) and immature neuron cells (Figure 4g,h) also increased at 4 months compared to that of the control VD rats. Besides, compared to H‐NSCs isolated from PBS group at each time point respectively, H‐NSCs isolated from ESC‐sEVs group exhibited higher proliferation (Figure 4i) and differentiation (Figure 4j) ability. These results revealed that ESC‐sEVs can restore cognitive of VD rats and this effect may be mediated by ameliorating the senescence of H‐NSCs.

### 2.3 ESC‐sEVs Rejuvenate Hippocampal NSCs Senescence and Enhanced NSCs Activity In Vitro

**Figure 5:**
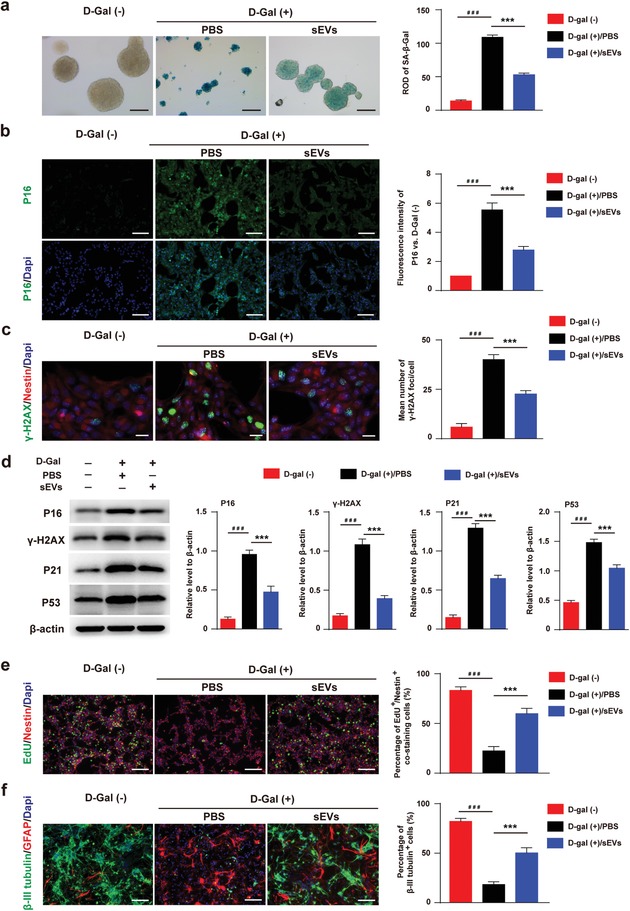
ESC‐sEVs ameliorate H‐NSCs senescence and reverse neurogenesis dysfunction induced by D‐gal in vitro. a) SA‐β‐gal staining and quantification of SA‐β‐gal intensity in neurospheres of D‐gal(−), and D‐gal(+) treated with ESC‐sEVs (D‐gal(+)‐sEVs) or PBS (D‐gal(+)‐PBS) group (Scale bar, 100 µm; *n* = 3/group; ***P < 0.001, ###*P* < 0.001). b) Immunofluorescence staining of p16^INK4a+^ (green) and quantification of the intensity in D‐gal(−), D‐gal(+)‐sEVs, and D‐gal(+)‐PBS group (Scale bar, 100 µm; *n* = 3/group; ***P < 0.001, ###*P* < 0.001). c) Immunofluorescence staining of γ‐H2AX^+^ (green) and quantification of the mean number of γ ‐H2AX foci per cell in D‐gal(−), D‐gal(+)‐sEVs, and D‐gal(+)‐PBS group (Scale bar, 20 µm; *n* = 3/group; ***P < 0.001, ###*P* < 0.001). d) Western blot analysis and quantification of p16^INK4a^, γ‐H2AX, P21, P53 in D‐gal(−), D‐gal(+)‐sEVs, and D‐gal(+)‐PBS group (*n* = 3/group; ***P < 0.001, ###*P* < 0.001). e) Immunofluorescence staining of EdU incorporation and quantification of EdU^+^/Nestin^+^/Dapi^+^cells in Nestin^+^/Dapi^+^ cells in D‐gal(−), D‐gal(+)‐sEVs, and D‐gal(+)‐PBS group (Scale bar, 100 µm; *n* = 3/group; ***P < 0.001, ###*P* < 0.001). f) Immunofluorescence staining of neuron differentiation and quantification of β‐III tubulin^+^ cells in whole cells in D‐gal(−), D‐gal(+)‐sEVs, and D‐gal(+)‐PBS group (Scale bar, 100 µm; *n* = 3/group; ***P < 0.001, ###*P* < 0.001).

We also established D‐gal induced senescence model of H‐NSCs in vitro to verify the function of ESC‐sEVs. As shown in Figure S5b (Supporting Information), ESC‐sEVs can be internalized by senescent H‐NSCs after incubation for 12 h. Then, SA‐β‐gal staining was conducted to detect the anti‐senescence ability of ESC‐sEVs. The results showed that SA‐β‐gal activity significantly increased after D‐gal treatment, while ESC‐sEVs treatment can reverse D‐gal induced increasing of SA‐β‐gal activity (**Figure** **5**a). Immunofluorescence staining for p16^INK4a^ and γ‐H2AX as well as western blot for p16^INK4a^, γ‐H2AX, P21, and P53 both showed that D‐gal treatment increased these protein levels which were decreased by the adding of ESC‐sEVs as compared to PBS group (Figure 5b–d). What's more, D‐gal treatment inhibited the proliferation and neuronal differentiation of H‐NSCs, while ESC‐sEVs stimulation enhanced their impaired proliferation and differentiation abilities (Figure 5e,f). These results further confirmed that H‐NSCs senescent phenotypes can be reversed by ESC‐sEVs.

### 2.4 ESC‐sEVs Rejuvenate Senescent H‐NSCs through Inhibiting mTORC1, Promoting TFEB Nuclear Translocation, Lysosome Level Resumption

**Figure 6:**
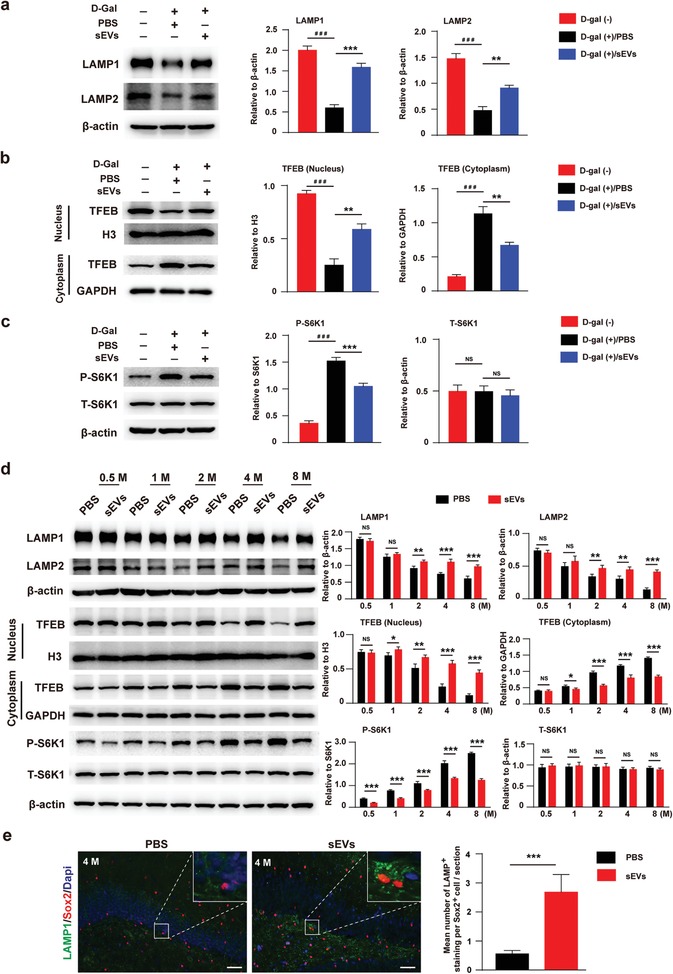
ESC‐sEVs inhibit mTORC1, promote TFEB nuclear translocation and lysosome level resumption in senescent H‐NSCs. Western blot analysis and quantification of a) LAMP1 and LAMP2, b) cytoplasmic TFEB and nuclear TFEB, c) P‐S6K1 and S6K1 in D‐gal(−), D‐gal(+)‐sEVs, and D‐gal(+)‐PBS group (*n* = 3; *P < 0.05, **P < 0.01, ***P < 0.001, ###*P* < 0.001). d) Western blot and quantification of LAMP1, LAMP2, nuclear TFEB, cytoplasmic TFEB, P‐S6K1, and S6K1 in isolated H‐NSCs in ESC‐sEVs and PBS group at each time point (*n* = 3; *P < 0.05, **P < 0.01, ***P < 0.001). e) Immunofluorescence staining of lysosome (LAMP1^+^, green) in H‐NSCs (Sox2^+^, red) and quantification of LAMP1^+^ in Sox2^+^ cells in ESC‐sEVs and PBS group at 4 months (Scale bar, 50 µm; *n* = 6/group; ***P < 0.001).

Evidence suggests that the lysosome level significantly reduced in aged NSCs, which resulted in the accumulation of protein aggregates and impairment of NSCs activation and neurogenesis, and restoration of lysosomal activity is sufficient to reactivate aged NSCs.^[^ ^30^ ^]^ Thus, we tested whether ESC‐sEVs could regulate lysosomal activity of senescent H‐NSCs. First, we examined the expression of lysosomal associated membrane protein 1 (LAMP1) and 2 (LAMP2) in D‐gal treated H‐NSCs. Western blot results showed that the level of these two proteins decreased in senescent H‐NSCs, while treatment with ESC‐sEVs increased the protein level of LAMP1 and LAMP2 (**Figure** **6**a). Next, we detected the expression pattern of transcription factor EB (TFEB), which is the most important factor in regulating lysosome biogenesis and functions by translocating into the nucleus to activate related‐genes.^[^ ^31^ ^]^ As shown in Figure S6a (Supporting Information), TFEB mostly located in cytoplasm in senescent H‐NSCs, after ESC‐sEVs treatment, TFEB translocated into nucleus. Western blot also confirmed the results (Figure 6b). TFEB nuclear translocation is regulated by mammalian target of rapamycin complex 1 (mTORC1), which enable the phosphorylation of TFEB and its retention in the cytoplasm.^[^ ^32^ ^]^ Therefore, we further detected mTORC1 activation as indicated by S6K1 phosphorylation in senescent H‐NSCs.^[^ ^33^ ^]^ We showed that the level of phosphorylated S6K1 (P‐S6K1) in ESC‐sEVs group was much lower than that in PBS group (Figure 6c). In order to demonstrate that inhibit mTORC1 activation can promote TFEB transfer from the cytosol to the nucleus in senescent H‐NSCs, we used rapamycin (25 × 10^−9^ m) to specifically inhibit mTORC signaling. As shown in Figure S6b,c (Supporting Information), the level of P‐S6K1 was significantly down‐regulated by rapamycin, and TFEB mostly located in nucleus in senescent H‐NSCs when treated with rapamycin. These results indicated that mTORC1 is activated in senescent H‐NSCs and TFEB was retained in the cytoplasm, and ESC‐sEVs increased TFEB nuclear translocation by inhibiting mTORC1 activation and promoted lysosome activation in senescent H‐NSCs.

We further detected the expression of these protein in isolated H‐NSCs from sham and VD groups, as well as PBS and ESC‐sEVs treated VD rats. The protein level of LAMP1, LAMP2, and nuclear TFEB decreased and mTORC1 was activated gradually in VD rats (Figure S7, Supporting Information), while in the H‐NSCs from ESC‐sEVs treated rats, this tendency was reversed (Figure 6d). The in vivo date also confirmed the resumption of lysosomes in the H‐NSCs of VD rats treated with ESC‐sEVs (Figure 6e). These results demonstrated that mTORC1 was activated in senescent H‐NSCs in VD rats in a time‐dependence manner, which leaded to the retention of TFEB in cytoplasm and reduction of lysosome. ESC‐sEVs rejuvenated H‐NSCs senescence in VD rats via inhibiting the activation of mTORC1, increasing TFEB nuclear translocation and lysosome resumption.

### 2.5 ESC‐sEVs Inhibit mTORC1 in Senescent H‐NSCs by transferring miRNAs

We further investigated the mechanism by which ESC‐sEVs inhibited mTORC1 in senescent H‐NSCs. miRNAs are one of the key molecules in sEVs to modulate recipient cells' function,^[^ ^34^ ^]^ thus, we used microarray to identify miRNAs in ESC‐sEVs and the result was listed in Table S3 (Supporting Information). We further searched the published studies (Literature search) to select candidate miRNA based on two requirements: first, mTORC1 activation inhibition effect; second, highly enrichment in ESC‐sEVs. As a result, miR‐17‐5p,^[^ ^35^ ^]^ miR‐18a‐5p,^[^ ^36^ ^]^ miR‐21‐5p,^[^ ^37^ ^]^ miR‐29a‐3p,^[^ ^38^ ^]^ and let‐7a‐5p^[^ ^39^ ^]^ were selected as target candidates. The levels of these five miRNAs in ESC‐sEVs were confirmed by RT‐qPCR (Figure S8a, Supporting Information).

**Figure 7:**
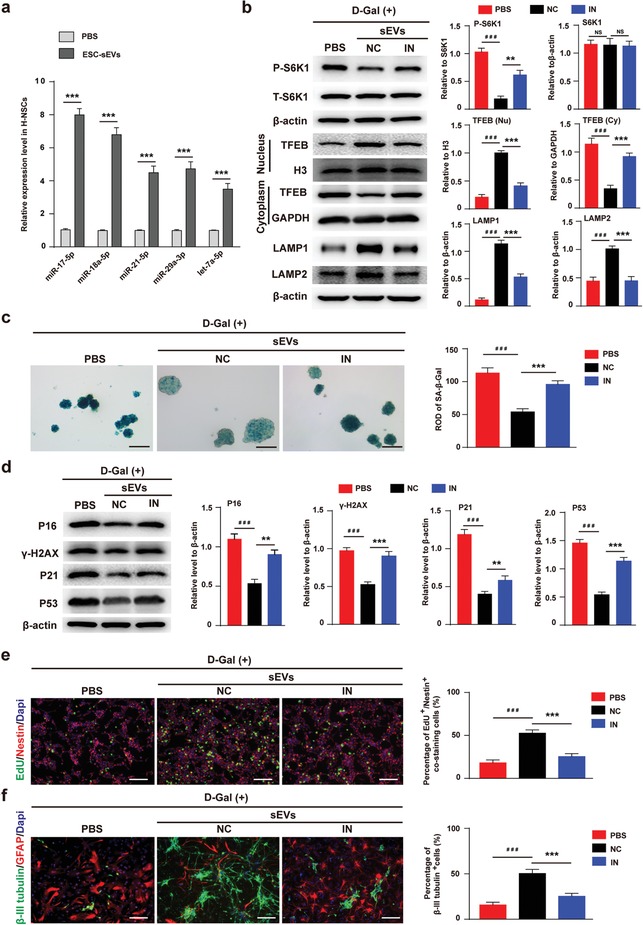
ESC‐sEVs transfer miRNAs to inhibit mTORC1 in senescent H‐NSCs. a) RT‐qPCR analysis identify the level of miR‐17‐5p, miR‐18a‐5p, miR‐21‐5p, miR‐29a‐3p, and let‐7a‐5p in senescent H‐NSCs after incubation with ESC‐sEVs for 3 passages (*n* = 3; ***P < 0.001). b) Western blot and quantification of P‐S6K1, S6K1, cytoplasmic TFEB, nuclear TFEB, LAMP1, and LAMP2 in D‐gal(+) H‐NSCs treated with PBS, ESC‐sEVs containing miRNAs‐negative control (ESC‐sEVs‐NC), and ESC‐sEVs containing miRNAs‐inhibitors (ESC‐sEVs‐IN) (*n* = 3; ***P < 0.001, ###*P* < 0.001). c) SA‐β‐gal staining and quantification of SA‐β‐gal intensity in neurospheres of PBS, ESC‐sEVs‐NC, or ESC‐sEVs‐IN group (Scale bar, 100 µm; *n* = 3/group; ***P < 0.001, ###*P* < 0.001). d) Western blot analysis and quantification of p16^INK4a^, γ‐H2AX, P21, and P53 in PBS, ESC‐sEVs‐NC, or ESC‐sEVs‐IN group (*n* = 3/group; **P < 0.01, ***P < 0.001, ###*P* < 0.001). e) Immunofluorescence staining of EdU incorporation and quantification of EdU^+^/Nestin^+^/Dapi^+^ cells in Nestin^+^/Dapi^+^ cells in PBS, ESC‐sEVs‐NC, or ESC‐sEVs‐IN group (Scale bar, 100 µm; *n* = 3/group; ***P < 0.001, ###*P* < 0.001). f) Immunofluorescence staining of neuron differentiation and quantification of β‐III tubulin^+^ cells in whole cells in PBS, ESC‐sEVs‐NC, or ESC‐sEVs‐IN group (Scale bar, 100 µm; *n* = 3/group; ***P < 0.001, ###*P* < 0.001).

We then evaluated whether ESC‐sEVs rejuvenate H‐NSCs senescence by transferring the five miRNAs to inhibit mTORC1 activation. At first, we showed ESC‐sEVs can transfer the five miRNAs and increased their expression level in senescent H‐NSCs (**Figure** **7**a). Then, the inhibitors of these five miRNAs were transferred into ESC‐sEVs to block their function. As shown in Figure S8b (Supporting Information), Dio‐labeled ESC‐sEVs (green) overlapped with Cy3‐labeled miRNA inhibitors (red) could be detected in cells, suggesting that miRNA inhibitors were successfully loaded into ESC‐sEVs and transferred to senescent H‐NSCs. After incubation with inhibitors‐containing ESC‐sEVs (IN group), the inhibitory function of ESC‐sEVs on mTORC1 activation was abolished, and the expression of nuclear TFEB was downregulated while the cytoplasmic TFEB was upregulated. The level of LAMP1 and LAMP2 in IN group was also much lower than that in NC group (Figure 7b). Moreover, the activity of SA‐β‐gal, as well as the expression of p16^INK4a^, γ ‐H2AX, P21, and P53 in neurospheres of IN group were much higher than in NC group (Figure 7c,d). Besides, compared to NC group, the proliferation and neuronal differentiation capacity of H‐NSCs in IN group decreased (Figure 7e,f). Overall, these results indicated that ESC‐sEVs transfer these mTORC1 targeting miRNAs to inhibit its activation in senescent H‐NSCs, then promote TFEB nuclear translocation and lysosome resumption, which resulted in senescent H‐NSCs rejuvenation, as well as self‐renewal and neurogenesis recovery.

## 3 Discussion

As one of the most common type of dementia, the pathogenesis of VD is still unclear, and until now there are no effective medications for the treatment of VD. In this study, we found a progressive cognitive dysfunction in VD rats accomplished with a time‐related increase of H‐NSCs senescence, H‐NSCs loss and neurogenesis decline. ESC‐sEVs treatment significantly ameliorated H‐NSCs senescence, maintained H‐NSCs number and neurogenesis, and reversed cognitive impairment. Furthermore, ESC‐sEVs exerted the anti‐senescence effects through transferring highly rich miRNA including miR‐17‐5p, miR‐18a‐5p, miR‐21‐5p, miR‐29a‐3p, and let‐7a‐5p to inhibit mTORC1 activation, promote TFEB nuclear translocation and lysosome resumption. To our knowledge, our data demonstrated for the first time that H‐NSCs senescence contributes to cognitive impairment in VD and ESC‐sEVs could alleviate H‐NSCs aging by lysosome activation. Targeting senescent H‐NSCs by ESC‐sEVs may represent a new therapeutic avenue for VD.

Hippocampal neurogenesis plays a crucial role in keeping and restoring of hippocampus‐dependent functions.^[^ ^4^ ^]^ The decreasing of hippocampal neurogenesis was accounted for age‐related cognitive decline as well as neurodegenerative disorders.^[^ ^5^, ^6^ ^]^ Restoring H‐NSCs and promoting their neurogenesis will increase the production of functional granule neurons and their integration into the existing hippocampal circuits, and this has been demonstrated to be conducive to damaged hippocampal structure and function repair.^[^ ^40^, ^41^ ^]^ In our study, we found H‐NSCs depletion and neurogenesis reduction were accomplished with the progress of VD, indicating that neurogenesis reduction in hippocampus is an important mechanism for cognition decline in VD. However, the intrinsic mechanism of H‐NSCs loss and neurogenesis reduction in VD is still unclear. Because cellular senescence is an important mechanism in cell functional impairment,^[^ ^7^ ^]^ and NSCs senescence limits their proliferation and inhibits neurogenesis.^[^ ^13^ ^]^ Besides, abnormal O_2_ levels, neuroinflammation, and oxidative stress in VD were important factors to induce cell senescence.^[^ ^17^ ^]^ Thus, we speculated H‐NSCs, the fundamental of neurogenesis, became senescence in VD. Several lines of evidence support this hypothesis, as the expression of p16^INK4a^ in H‐NSCs was much higher in VD rats, the intensity of SA‐β‐gal and the expression of p16^INK4a^, γ ‐H2AX, P21, P53 in isolated H‐NSCs neurospheres were increased in a time‐related manner. Moreover, H‐NSCs isolated from VD rats showed a time‐related proliferation and neuron differentiation impairment. All of these results indicated that acceleration of H‐NSCs senescence may be the intrinsic mechanism for H‐NSCs loss and neurogenesis decline in the process of VD.

ESCs are cells derived from the early embryo that can be propagated infinitely in the primitive undifferentiated state while remaining pluripotent, which indicated an excellent cellular source for the treatment of aging‐related diseases.^[^ ^22^ ^]^ Min et al. had demonstrated that transplantation of ESCs could improve myocardial function in aging rats through angiogenesis and myogenesis.^[^ ^42^ ^]^ As Adamiak et al. had demonstrated Induced pluripotent stem cell (iPSCs)‐derived sEVs are more effective for cardiac repair than iPSCs.^[^ ^43^ ^]^ Thus, we believe ESC‐sEVs could be powerful in treating cellular aging and aging‐associated diseases. Recently, ESCs‐sEVs had been demonstrated to ameliorate somatic cell senescence to promote tissue recovery.^[^ ^23^, ^24^ ^]^ However, whether ESC‐sEVs can rejuvenate senescent stem cells and recover their capacity is still unknown. Rejuvenation of stem cells to enhance their self‐renewal and regeneration capacity is more conducive to functional recovery.^[^ ^8^ ^]^ For example, Sinha et al.^[^ ^44^ ^]^ argued that methods to rejuvenate senescent muscle satellite stem cells will reverse their functional impairments, restore genomic integrity, improve muscle structural and functional features, and increase strength and endurance exercise capacity. Thus, rejuvenating senescent H‐NSCs to reverse senescence‐associated neurogenesis dysfunction seem to be a promising therapeutic approach for VD. In our study, we found chronic ESC‐sEVs treatment could reduce the senescence hallmark, recover the compromised self‐renewal and neurogenesis capacity of H‐NSCs in VD rats, and result in cognitive recovery. These results means the therapeutic effects of ESC‐sEVs on VD may be attributed to rejuvenate H‐NSCs senescence.

Lysosomes are acidic organelles that play critical roles in maintaining cellular homeostasis by digesting and recycling the majority of cellular macromolecules.^[^ ^45^ ^]^ Lysosomes are important in senescence regulation for they can deplete total histone content like γ‐H2AX in senescent cells to contribute to cell stability.^[^ ^46^ ^]^ Then, for the first time we showed the ESC‐sEVs could increase lysosomes level in senescent H‐NSCs. According to a recent research,^[^ ^30^ ^]^ lysosomes are important contributor in regulating NSCs senescence, their decrease in senescent NSCs resulted in the accumulation of protein aggregates and impairment of NSCs activation and neurogenesis. TFEB is the most important regulator in the process of lysosome biogenesis and functions,^[^ ^31^ ^]^ and TFEB nuclear translocation is regulated by mTORC1, which enable to phosphorylate TFEB and retain it in cytoplasm. In our study, we found a down‐regulation of nuclear TFEB and up‐regulation of mTORC1 in senescent H‐NSCs, and ESC‐sEVs can reverse these changing. As noted above, ESC‐sEVs rejuvenate senescent H‐NSCs partly by inhibiting mTORC1 activation, promoting TFEB nuclear translocation and lysosome resumption. However, how ESC‐sEVs inhibit mTORC1 activation still uncovered.

Recently, many researches have demonstrated sEVs act as a delivery system partly by transferring miRNAs to recipient cells to alter their gene expression and bioactivity.^[^ ^34^ ^]^ In addition, miRNAs are important in regulating NSCs function^[^ ^47^ ^]^ as well as cognition and aging.^[^ ^48^ ^]^ Therefore, we further detected the variety and expression level of miRNAs in ESC‐sEVs. We found miR‐17‐5p, miR‐18a‐5p, miR‐21‐5p, miR‐29a‐3p, and let‐7a‐5p are highly‐expressed in ESC‐sEVs and involved in mTORC1 inhibition. Further study demonstrated that upon the five miRNAs in ESC‐sEVs was blocked by their inhibitors, ESC‐sEVs almost failed to prevent the activation of mTORC1 and to promote TFEB nuclear translocation as well as lysosome resumption. The rejuvenative effect of ESC‐sEVs on senescent H‐NSCs was also abolished, although not completely. These findings suggested that miR‐17‐5p, miR‐18a‐5p, miR‐21‐5p, miR‐29a‐3p, and let‐7a‐5p are crucial mediators in ESC‐sEVs induced rejuvenation of senescent H‐NSCs. However, it should be noted that the effects of ESC‐sEVs on mTORC1 inhibition and H‐NSCs senescence were not entirely abolished by miRNAs blocked in ESC‐sEVs, suggesting the presence of additional miRNAs may be involved in these processes. As list in Table S3 (Supporting Information) of microarray, miRNAs like miR‐211‐3p,^[^ ^49^ ^]^ miR‐214‐3p,^[^ ^50^ ^]^ miR‐184,^[^ ^51^ ^]^ and miR‐99a‐5p,^[^ ^52^ ^]^ which can inhibit mTORC1 activation, were also contained in ESC‐sEVs though in low level and may function to rejuvenate H‐NSCs senescence. In addition, as we previously demonstrated ESC‐sEVs can transfer highly enriched miR‐200a to ameliorate endothelial senescence by upregulation of Nrf2,^[^ ^24^ ^]^ while whether miR‐200a in ESC‐sEVs can function in senescent H‐NSCs still needs further exploration.

In summary, our data demonstrated for the first time that H‐NSCs senescence resulted in H‐NSCs depletion and neurogenesis reduction, and finally caused cognitive dysfunction in VD. ESC‐sEVs rejuvenate senescent H‐NSCs partly by transferring their highly rich miR‐17‐5p, miR‐18a‐5p, miR‐21‐5p, miR‐29a‐3p, and let‐7a‐5p to inhibit mTORC1 activation and promote TFEB nuclear translocation, and contribute to lysosome resumption. Thus, the application of ESC‐sEVs may be a novel cell‐free therapeutic tool for patients with VD, as well as other aging‐related diseases.

## 4 Experimental Section

Details of materials and experimental procedures are available in the Supporting Information.

##### ESC Culture, ESC‐sEVs Isolation and Identification

The human ESCs (H9) were provided by the Institute of Biochemistry and Cell Biology of Chinese Academy of Sciences (Shanghai, China), and were cultured in ncEpic hPSC Medium (Nuwacell Biotechnologies, China). ESC‐sEVs were isolated by differential ultracentrifugation protocols. The morphology of ESC‐sEVs was observed by transmission electron microscope (TEM; JEM 1400, Tokyo, Japan). The size distribution and particle concentration of ESC‐sEVs were measured using the nanoflow cytometer (N30 Nanoflow Analyzer; NanoFCM Inc., Xiamen, China). Markers such as CD9, CD63, TSG‐100, and GM130, β‐actin, Lamin A/C, were analyzed by western blot. Further details are provided in the Supporting Information.

##### Animal Experimental Procedures

Permanent occlusion of the bilateral common carotid artery (BCCAO) model was applied in the study, which can imitate the pathological change with significant injury in the white matter and hippocampal neuronal damage in rats of VD.^[^ ^2^ ^]^ Healthy adult male Sprague Dawley rats (≈8 weeks old) weighing 250–300 g were obtained from Shanghai Slack Laboratory Animal Co. Ltd. (Shanghai, China). Animal care and experimental procedures were approved by the Animal Research Committee of the Sixth People's Hospital at the Shanghai Jiao Tong University (SYXK [Shanghai, China] 2011‐0128, 1 January 2011). These rats were randomly assigned to sham group (sham‐operated group, *n* = 50) and VD group (*n* = 50), as well as PBS group (VD + PBS; *n* = 50) and ESC‐sEVs group (VD + sEVs; *n* = 50). Further details of BCCAO model are provided in the Supporting Information. ESC‐sEVs (1 × 10^10^ particles per 200 µL) and sterile PBS (200 µL) were given to rats via intravenously injection, as systemic injection of sEVs can accumulate in brain to exert their therapeutic effects.^[^ ^53^ ^]^ Injection began at the second day after surgery, and thereafter once every 2 days in the first week and once per week in the later successive weeks until sacrifice at different time points (0.5, 1, 2, 4, and 8 months). Rats were injected with 50 mg kg^−1^ EdU 3 days before sacrificing to label proliferated cells, and sacrificed for EdU analyzation at 4 months.

##### Morris Water Maze

Morris water maze (MWM) test was employed to assess spatial memory and learning abilities of rats, details are described in the Supporting Information.

##### Immunofluorescence Staining and Western Blot Analysis

Details for immunofluorescence staining and western blot analysis are provided in the Supporting Information.

##### Senescence‐Associated β‐galactosidase Staining

Senescence‐associated β‐galactosidase (SA‐β‐gal) staining of brain sections or neurospheres was performed using the SA‐β‐gal staining kit (Beyotime Biotechnology). The intensity of SA‐β‐gal positive cells was evaluated by means of a ROD (relative optical density) value. Further details are provided in the Supporting Information.

##### NSCs Isolation and Cultivation, Proliferation, and Differentiation Assay

NSCs were isolated from adult rats' hippocampus as described previously.^[^ ^54^ ^]^ Neurospheres formation and EdU incorporation experiment were used to detect NSC proliferation ability. Neuron differentiation assay was used to detect NSC differentiation ability. Further details are provided in the Supporting Information.

##### Effects of ESC‐sEVs on NSCs Senescence

D‐galactose (D‐gal) induced aging model is a systemic and homogeneous aging model,^[^ ^55^ ^]^ it was applied to induce NSCs senescence in vitro as described before.^[^ ^56^ ^]^ Senescent NSCs were incubated with 1 × 10^10^ particles per mL ESC‐sEVs or an equal volume of PBS for 3 passages. ESC‐sEVs uptake assay, NSCs senescence detection, proliferation and differentiation assay are described in the Supporting Information.

##### ESC‐sEVs miRNA Expression Profiling and Real‐Time Quantitative Polymerase Chain Reaction (RT‐qPCR) Analysis

Microarray analysis was performed on Agilent Human miRNA 8 × 60K format v21.0 (based on Sanger miRbase version 21.0) by Genomax Technologies. For RT‐qPCR assay, ESC‐sEVs miRNAs were isolated by using the miRNeasy Mini Kit (Qiagen). The reverse transcription reactions of miRNAs were performed using the miScript II RT Kit (Qiagen). The PCR reaction was carried out with the ABI Prism 7900HT Real Time System (Applied Biosystems) by using the miScript SYBR Green PCR Kit (Qiagen) and miScript Primer Assay(Qiagen). The miScript Primer Assays for the target miRNA used are listed in Table S1 (Supporting Information). Further details are provided in the Supporting Information.

##### Deliver miRNA Inhibitor to ESC‐sEVs and Treating NSC

The miRNA inhibitors to miR‐17‐5p, miR‐18a‐5p, miR‐29a‐3p, miR‐21‐5p, let‐7a‐5p, and Negative Control were obtained from GenePharma (Shanghai, China). The sequences of these inhibitors are listed in Table S2 (Supporting Information). Exo‐FectTM siRNA/miRNA Transfection Kit (System Biosciences) was used to deliver miRNA inhibitors into ESC‐sEVs. Further details are provided in the Supporting Information.

##### Statistical Analysis

All data were presented as mean ± SEM. Student's *t*‐test was employed to examine the inter‐group differences, whereas one‐way analysis of variance (ANOVA) were utilized to explore the heterogeneity among different groups, followed by Bonferroni post hoc test in the absence of equivalent variance. A difference of *P* < 0.05 was deemed to be statistically significant.

## Conflict of Interest

The authors declare no conflict of interest.

## Author Contributions

G.H. and Y.X. contributed equally to this work. G.W.H. and Y.G.X. performed the experiments, collected and analyzed the data and prepare the manuscript, J.T.Z. performed animal experiments, Y.C. and J.Y. performed immunostaining, X.N. performed ESC‐sEVs isolation and identification, B.Z.Z. give financial support, Q.L., Y.W., and Z.F.D. conceptualized the project, provided funding, supported study material, and final approved the manuscript.

## Supporting information

Supporting Information

Supporting Information
