# Supplementary material for: ESC‐sEVs Rejuvenate Senescent Hippocampal NSCs by Activating Lysosomes to Improve Cognitive Dysfunction in Vascular Dementia
Source: Adv Sci (Weinh). 2020 Mar 20;7(10):1903330. doi: 10.1002/advs.201903330 (PMC7237844; doi:10.1002/advs.201903330)
Supplement: Supplementary file 1 — Supporting Information [file ADVS-7-1903330-s001.pdf]

Copyright WILEY-VCH Verlag GmbH & Co. KGaA, 69469 Weinheim, Germany, 2020.

ADVANCED SCIENCE
Open Access

Supporting Information

for *Adv. Sci.,* DOI: 10.1002/advs.201903330

ESC-sEVs Rejuvenate Senescent Hippocampal NSCs by Activating Lysosomes to Improve Cognitive Dysfunction in Vascular Dementia

Guowen Hu, Yuguo Xia, Juntao Zhang, Yu Chen, Ji Yuan, Xin Niu, Bizeng Zhao, Qing Li, Yang Wang,* and Zhifeng Deng**

SUPPLEMENTAL MATERIAL

1. Materials and methods

ESC culture

The human embryonic stem cells (ESC; H9) were provided by the Institute of Biochemistry and Cell Biology of Chinese Academy of Sciences (Shanghai, China). The ESC were cultured in Nuwacell™ hiPSC/hESC medium (NO. RP01001; Nuwacell Biotechnologies, China) on a Nuwacell™-Vitronectin (NO. RP01002-A; Nuwacell) coated plate under the standard conditions of 5%CO2 and 37°C in humidified incubator. The medium was changed daily and cells were passaged at 80~90% confluence. ESCs identification was performed by ALP staining and immunofluorescence staining for markers (Nanog, OCT4, SSEA4, TRA-1-60, and TRA-1-81) in the ESC colonies.

ESC-sEVs isolation and identification

ESC-sEVs isolation

ESC-sEVs was isolated by differential ultracentrifugation protocols from ESCs condition medium (ESC-CM) according to the MISEV2018 guideline [1]. Briefly, ESC-CM was centrifuged at 300×g for 10 min to remove dead cells and 2000×g for 20 min to remove cellular debris and apoptotic bodies. Then, the supernatant was centrifuged at 10,000×g for 30 min and filtered through 0.22 µm sterilized filter (Millipore, Bedford, MA, USA) to remove large EVs. Next, the supernatant was subjected to ultracentrifugation at 100,000×g for 114 min by using a SW 32 Ti Rotor Swinging Bucket rotor (K factor of 256.8, 28536 rpm; Beckman Coulter, Fullerton, CA) to pellet

ESC-sEVs. After removing the supernatant, the ESC-sEVs pellet was washed with PBS and followed by a second ultracentrifugation at 100,000×g for 114 min. All centrifugation steps were performed at 4°C. Finally, the pellet was re-suspended in PBS and stored at -80°C.

Transmission Electron Microscope (TEM)

The morphology of ESC-sEVs was observed by TEM (JEM 1400, Tokyo, Japan). Briefly, a total of 10μL ESC-sEVs enriched solution was placed on a formvar-carbon coated grid (300 meshes) and left to dry at room temperature for 20min. Then, ESC-sEVs was washed with PBS and fixed in 1% glutaraldehyde for 5 min. Next, ESC-sEVs was washed with water and stained with saturated aqueous uranyl oxalate for 5 min. Finally, the grid was dried at room temperature for 10 min and then imaged.

Size distribution and particle concentration

The size distribution and particle concentration of ESC-sEVs were measured by using the nano-flow cytometer (N30 Nanoflow Analyzer, NanoFCM Inc., Xiamen, China) as described previously [2]. Briefly, the side scatter intensity (SSI) was measured by the loading of the standard polystyrene nanoparticles (200 nm) with a concentration of 1.58 $\times 10^8$/mL to the nano-flow cytometer. Next, isolated ESC-sEVs sample diluted with 1000-fold PBS (for a nanoparticle concentration of approximately 5×10^9/mL) was loaded to the nano-flow to measure the SSI. Finally, The concentration of EVs was calculated according to the ratio of SSI to particle concentration in the standard polystyrene nanoparticles. For size measurement, standard silica nanoparticles with mixed size (68nm, 91nm, 113nm, 155nm) were load to the nano-flow cytometer to

generate a standard cure, followed by the loading of sEVs sample. The size distribution was calculated according to the standard cure.

Protein concentration

The protein concentration of ESC-sEVs was quantified by Pierce BCA Protein Assay Kit (Thermo Scientific) according to the product manual. Briefly, 200 μL of the WR solution was loaded into each well of a 96-well plate. Next, 10 μL of the ESC-sEVs sample was added. Finally, the plate was incubated at 37°C for 30 min and the absorbance was detected at 562 nm. A standard curve was used to determine the protein concentration of each ESC-sEVs sample.

Particle parameters measurement

ESC-sEVs were isolated by differential ultracentrifugation protocols from ESCs condition medium (ESC-CM) according to the MISEV2018 guideline (J Extracell Vesicles. 2018; 7(1): 1535750). To calculate the ESC-sEVs parameters, we firstly cultured 10 dishes of ESC-sEVs with fresh medium for one day, then collected and quantified the ESCs and ESC-CM. ESC-CM was used to isolate ESC-sEVs by differential ultracentrifugation protocols and the size distribution and particle concentration of ESC-sEVs were measured using the nano-flow cytometer (N30 Nanoflow Analyzer, NanoFCM Inc., Xiamen, China). The protein concentration of ESC-sEVs was quantified by Pierce BCA Protein Assay Kit. From these procedures, we got the total particle number of ESC-sEVs (a), the total protein quantity of ESC-sEVs (b), the total volume of ESC-CM (c), and the total number of ESCs (d). Next, we calculated the mean particle concentration in CM (a/c) , particle number per cell

(a/d), the mean protein concentration of ESC-sEVs in CM (b/c) and per particle (b/a). We used 6 EV samples per trial and 6 trials were conducted in total.

Western blot

The expression of sEVs markers such as CD9, CD63, and TSG-101 was analyzed by western blot. GM130, β-actin, and Lamin A/C were also detected to determine the purity of ESC-sEVs. Briefly, the ESC-sEVs pellet was routine ultracentrifugation as described above. ESC-sEVs protein was harvested by using RIPA lysis buffer supplemented with protease inhibitor cocktail (Roche). Then, the protein concentration of ESC-sEVs was detected by using the Pierce BCA Protein Assay Kit as described above. Next, protein was separated by sodium dodecyl sulfatepolyacrylamide gel electrophoresis and transferred to polyvinylidene fluoride membranes (Millipore). The membranes were blocked with 5% non-fat milk for 1 h and incubated overnight at 4 °C with the following antibodies: rabbit monoclonal anti-β-actin (1:1,000; Abcam, NO. ab179467), rabbit monoclonal anti-CD9 (1:1,000; Abcam, NO. ab92726), rabbit monoclonal anti-CD63 (1:1,000; Abcam, NO. ab134045), mouse monoclonal anti-TSG-101 (1:1,000; Abcam, NO.ab83), mouse polyclonal anti-GM130 (1:500; Abcam, NO.ab169276), Rabbit polyclonal anti-Lamin A/C (1:1000; Servicebio, NO. GB11407). Membranes were then incubated with HRP-conjugated secondary antibodies (1:3,000; Cell Signaling Technology) at room temperature for 1 h. The immunoreactive bands were visualized using ECL (Thermo Fisher Scientific) and imaged with a FluorChem M Fluorescent Imaging System (ProteinSimple, Santa Clara, CA, USA).

Animal Experimental Procedures

Permanent occlusion of the bilateral common carotid artery (BCCAO) model was applied in the study, which can imitate the pathological change with significant injury in the white matter and hippocampal neuronal damage in rats of VD[3]. Healthy adult male Sprague Dawley rats (approximately 8 weeks old) weighing 250-300 g were obtained from Shanghai Slack Laboratory Animal Co. Ltd. (Shanghai, China), and housed in a specific pathogen-free (SPF) animal laboratory. Animal care and experimental procedures were approved by the Animal Research Committee of the Sixth People's Hospital at the Shanghai Jiao Tong University (SYXK [Shanghai, China] 2011-0128, 1 January 2011). These rats were randomly assigned to sham group (sham-operated group, n = 50), VD group (BCCAO-operated group, n = 50), PBS group (BCCAO-operated + PBS; n = 50), and ESC-sEVs group (BCCAO-operated + sEVs; n = 50).

Bilateral common carotid arteries were occluded as described previously [4]. Briefly, rats were anesthetized by intraperitoneal injection of 50 mg/kg pentobarbital sodium (Sigma-Aldrich), Then a ventral midline incision (1.5–2.0 cm) was made to expose both carotid arteries, and the arteries were gently isolated from the surrounding tissues such as the vagal nerves and carotid sheath. 4-0 silk sutures were used to double ligate the the artery. After that, the skin incision was closed with normal suture. Sham group rats were subjected to the same surgical procedure without ligation of the arteries. The entire surgical procedure was performed under sterile conditions, and the body temperature was regulated by a heating lamp within the range of 37 ± 1°C. ESC-sEVs (1×10^{10} particles/200 μL) and sterile PBS (200 μL) were given to rats via intravenously

injection beginning at the second day after surgery, and thereafter once every two days in the first week and once per week in the later successive weeks until sacrifice at different time points (0.5M, 1M, 2M, 4M, 8M).

Rats were injected with 5-ethynyl-2′-deoxyuridine (EdU, Invitrogen, in 0.9% saline, 0.007 N NaOH) in a concentration of 50 mg/kg 3 days before sacrificing to label the proliferated cells. Rats were sacrificed and analyzed for EdU at the point of 4M after surgery.

In vivo ESC-sEVs migration into brain

To determine the migration of ESC-sEVs into the brain, ESC-sEVs were labeled with the Molecular Probes' Vybrant (ThermoFisher Scientic) 1,1'-dioctadecyl-3,3,3',3'-tetramethylindotricarbocyanine iodide (DiR) according to the protocol as previously described with small modification [5]. Briefly, ESC-sEVs were incubated with DiR fluorescent dye under room temperature for 15 minutes followed by washed with PBS and ultracentrifugation for three times to get rid of the unlabeled dye. VD rats (n = 3) at 12h post-surgery were intravenously administered with a single dose of near-infrared uorescent dye DiR-labeled ESC-sEVs (almost 1×10^{11} particles in 200 μL). The uorescence images of rats' brain were then recorded by the IVIS Spectrum/CT imaging system (Perkin-Elmer, USA) at 6h after the injection.

Morris Water Maze

Morris water maze (MWM) test was employed to assess spatial learning and memory abilities of rats as described previously [6]. The latency to escape onto the platform was recorded as the performance of spatial learning. Rats were trained once a day over four

consecutive days. In each trial, rats were gently released into the water with their head facing opposite of the platform, and were given a maximum of 90s to find the submerged platform. For rats that could not find the platform within 90s, they were guided to stay on it for 15s, and the score of 90s was given to such rats. To assess spatial memory, a spatial probe trial was performed on day 5 of the training trial. The platform was removed and rats were placed in water opposite to the target quadrant, and allowed to swim freely for 90s. The percentage of time that rats spent in the target quadrant within 90s was recorded. The Shanghai Xinran Mdt InfoTech Ltd (Shanghai, China) SuperMaze animal behavior record and analysis system was used for data collection and analysis.

Immunofluorescence staining

Rats were anesthetized and perfused transcardially with saline, then followed by 4% paraformaldehyde (PFA; 4.3 g/L NaOH, 40 g/L paraformaldehyde, 18.8 g/L NaH2PO4.H20). The brains were removed and post-fixed overnight in 4% PFA, and then dehydrated in 20% and 30 % sucrose at 4 °C, respectively. Each brain was embedded in OCT and cut every sixth 20-μm-thick coronal sections on a freezing microtome (Leica CM 1950; Leica Biosystem, Heidelberg, Germany). Cells were fixed by 4% PFA for 15 min. Brain sections or cultured cells were incubated with 0.3% Triton X-100 for 30 min and 5 % bovine serum albumin (BSA; Sigma, St. Louis, MO, USA) for 1 h, and then incubated overnight at 4 °C with the following antibodies: rabbit polyclonal anti-Sox2 (1:100; Cell Signaling Technology, NO. cst2748), mouse monoclonal anti-GFAP (1:100; Abcam, NO. ab10062), rabbit monoclonal anti-β-

Tubulin III (1:100; Abcam, NO.ab18207), mouse monoclonal anti-Nestin (1:100; Abcam, NO.6142), rabbit polyclonal anti-DCX (1:100; Abcam, ab18723), mouse monoclonal anti-LAMP1 (1:50; Santa Cruz, NO.sc-20011), rabbit monoclonal anti-Phospho-Histone H2A.X (Ser139) (γ-H2AX; 1:200; Cell Signaling Technology, NO. cst9718), mouse monoclonal anti- $p16^{INK4a}$ (1:200; Invitrogen, NO.MA5-17142), rabbit polyclonal anti-Nanog (1:100; Abcam, NO.ab109250), rabbit polyclonal anti-Oct4 (1:100; Abcam, NO.ab19857), mouse monoclonal anti-SSEA4 (1:100; Abcam, NO.ab16287), mouse monoclonal anti-TRA-1-60 (1:100; Abcam, NO.ab16288), mouse monoclonal anti-TRA-1-81 (1:100; Abcam, NO.ab16289), rabbit polyclonal anti-TFEB (1:1000; Bioss, NO.bs-5137R). Followed by three times of rinse in 0.1 M PBS (pH 7.4) before the incubation with secondary antibodies (1:400; Invitrogen) including Alexa Fluor® 594 goat anti-rabbit or anti-mouse IgG (H+L) or Alexa Fluor® 488 goat anti-rabbit or anti-mouse IgG (H + L) for 1 h at room temperature, protected from light. EdU^+ cells were stained by using Click-iT Edu Alexa Fluor 488 Imaging Kit (Life Technologies) following the manufacturer's protocol. Nuclei was visualized by using 2 μM DAPI (Sigma). Images were acquired using the Leica DM6B microscope (Leica Microsystems, Milan, Italy). The number or mean fluorescence intensity of positively stained cells were performed using the Image J software (National Institutes of Health, Bethesda, MD, USA).

Senescence-associated β-galactosidase staining

Senescence-associated β-galactosidase (SA-β-gal) staining of brain sections or neurospheres were performed using the SA-β-gal staining kit (Beyotime

Biotechnology). According to manufacturer's protocol, brain sections or cell cultures were fixed and then stained with SA-β-gal staining solution for 16-18 h at 37 °C (without CO_2), ice-cold PBS was used to stop the enzymatic reaction. Images of brain sections were acquired using the Leica DM6B microscope, and at least six images of neurospheres were acquired using the phase-contrast microscope (Leica Microsystems). The intensity of SA-β-gal positive cells was evaluated by means of a ROD (relative optical density) value. ROD of SA-β-gal positive cells was obtained after transformating mean gray values (obtained by Image Pro Plus software) into ROD via the formula: ROD = log (256/mean gray).

H-NSCs isolation and cultivation

H-NSCs were isolated from adult rats hippocampus as described previously [7]. Briefly, hippocampus was separated from brain and minced by scissors, then treated with enzyme cocktail solution at 37°C for 1 hr. Later mixed with an equal volume of percoll solution and centrifuged at 20,000 g for 30 min at 18°C. H-NSCs located in the lower layer fraction were harvested and washed three times with DMEM/ F12, and resuspended in complete H-NSCs medium: DMEM/F12 medium supplemented with 2% B27 (Gibco Life Technologies), 1% penicillin/streptomycin (Gibco Life Technologies), 20 ng/ml epidermal growth factor (EGF, ProSpec), 20 ng/ml basic fibroblast growth factor (bFGF, ProSpec), and 5 цg/ ml heparin (Sigma-Aldrich). Cells were seeded in a culture plate and the medium was fifty percent replaced every 2 days. In the ex vivo study, H-NSCs were isolated from VD rats at different time point, while in the in vitro study, H-NSCs were isolated from 2 weeks old rats.

In vitro ESC-sEVs uptake assay

ESC-sEVs were labeled with fluorescent carbocyanine dye (Dio, Life Technologies) for 30 min at 37 °C as our previously described [8]. The labeled ESC-sEVs were washed with PBS and pelleted by differential ultracentrifugation for three times. H-NSCs were incubated with Dio-labeled ESC-sEVs (1×10^{10} particles/mL) for 12 h. After that, culture medium was discarded and the cells were rinsed twice with PBS. H-NSCs were fixed, nucleus was stained with DAPI. Images were acquired using the Leica DM6B microscope.

Effects of ESC-sEVs on H-NSCs senescence

In the ex vivo study, neurospheres isolated from VD rats in passage two were dissociated with Accutase (Sigma-Aldrich). 20,000 cells were plated into individual wells of ultralow-binding 24-well plates and incubated in complete NSCs medium for 5 days. In the in vitro study, D-gal was used to induce H-NSCs senescence as described previously [9]. Briefly, H-NSCs in passage two was treated with 10 mg/mL D-gal for two passages to induced senescence. Then, senescent H-NSCs were incubated with 1×10^{10} particles/mL ESC-sEVs or an equal volume of PBS for three passages. Neurospheres were collected for experiments like SA-β-gal staining and western blot for senescence detection.

Effects of ESC-sEVs on H-NSCs proliferation and differentiation

In the ex vivo study, neurospheres isolated from VD rats in passage two were used for proliferation and differentiation assay. For the proliferation assay, neurospheres were dissociated and 20,000 cells were plated into individual wells of ultralow-binding 24-

well plates and incubated in complete NSCs medium for 5 days. The size (diameter) of neurospheres were counted under the phase-contrast microscope, and at least six images of neurospheres were acquired. In EdU proliferation assay, 20,000 cells were incubated in complete NSCs medium for 4 days and then administered EdU (10uM) for 4 hours. Neurospheres were dissociated and seeded into individual wells of 96-well plates and adhered for 4 hours. Then, H-NSCs were fixed and immunofluorescence staining to calculate the percentage of EdU^+ cells in whole cells. For the differentiation assay, neurospheres were dissociated and 50,000 cells were plated on poly-L-lysine (Sigma-Aldrich) coated 48-well plates. Cells were cultured with the differentiation medium: neural basal (NB) medium (Gibco Life Technologies) supplemented with 2% B27 and 1% fetal bovine serum (Gibco Life Technologies), and cultured for 5 days, and the medium was half-changed every other day. Cells were fixed and immunofluorescence staining for β-Tubulin III and GFAP to calculate the percentage of β-Tubulin III^+ cells in whole cells.

ESC-sEVs miRNA expression profiling

Microarray analysis was performed on Agilent Human miRNA 8x60K format v21.0 (based on Sanger miRbase version 21.0) by Genomax Technologies. Each array contained probes interrogating 2549 human microRNAs. Briefly, extracted microRNA was labeled and hybridized using the miRNA Complete Labeling and Hybridization Kit from Agilent, in accordance with the manufacturer's protocol (Agilent Technologies, Santa Clara, California, USA). After hybridization for 20 hours at 55 °C, the microarray was washed twice by the Gene Expression Wash Buffer kit (Agilent).

Hybridization signals were scanned on Agilent Microarray Scanner (Agilent) using Agilent scan control software Version A7.0 (Agilent). Data collection, background subtraction and array normalization were performed by Agilent Feature Extraction Software (Agilent). Percentile normalization and principal component analysis were performed by Gene spring 12.0 (Agilent).

Real-time quantitative polymerase chain reaction (RT-qPCR) analysis

ESC-sEVs miRNAs were isolated by using the miRNeasy Mini Kit (Qiagen) according to the manufacturer's protocol. In brief, ESC-sEVs were washed and centrifuged using Buffer XBP and XWP, and mixed with QIAzol lysate. Then, chloroform was added to separate the lysate, the upper aqueous phase was transferred to a new collection tube and added 2 volumes of 100% ethanol, after centrifugation, washed with Buffer RWT and RPE, as well as added DNase/RNase-Free water and centrifuged at 12,000×g for 1 min, the flow-through collected was the ESC-sEVs total RNA. The concentration and purity of RNA samples were detected by NanoDrop2000 spectrophotometer (ThermoFisher Scientific).

The reverse transcription reactions of miRNAs were performed using the miScript II RT Kit (Qiagen). Briefly, the reaction mixture of 20 μl contained 4 μl of 5x miScript Hispec Buffer, 2 μl of 10x miScript Nucleics Mix, 2 μl of miScript Reverse Transcriptase Mix, and 12 μl of RNase–free water with 200 ng RNA template. Thermal Cycling conditions are 37°C for 60 min and 95°C for 5 min.

The PCR reactions were carried out with the ABI Prism 7900HT Real Time System (Applied Biosystems, Carlsbad, CA) by using the miScript SYBR Green PCR Kit

(Qiagen) and miScript Primer Assay(Qiagen). The following RT-qPCR protocol was used: initial activation of HotStar-Taq DNA Polymerase (95°C, 15 min); 40 cycles of denaturation (94°C, 15 s), annealing (55°C, 30 s), and extension (70°C, 30 s). The miScript Primer Assays for the target miRNA we used are listed in Supplemental Table 1. The data was analyzed using the cycle threshold (Ct) value. Each experiment was performed in triplicate.

Delivery of miRNA inhibitor to ESC-sEVs and H-NSCs treatment

miRNA inhibitors are the exact antisense copy of the mature miRNA. The miRNA inhibitors to miR-17-5p, miR-18a-5p, miR-29a-3p, miR-21-5p, let-7a-5p, and Negative Control were obtained from GenePharma (Shanghai, China), where all the nucleotides in the inhibitors contain 2′-O-Me modifications at every base and a 5′-CY3 containing amino linker. The sequences of these inhibitors are listed in Supplemental Table 2. Exo-FectTM siRNA/miRNA Transfection Kit (System Biosciences) was used to deliver miRNA inhibitors into ESC-sEVs in accordance with the manufacturer’s instructions. Then, senescent H-NSCs were treated with different conditions: (1) senescent H-NSCs treated with PBS, (2) senescent H-NSCs treated with ESC-sEVs containing miRNA inhibitor Negative Control, and (3) senescent H-NSCs treated with ESC-sEVs containing miRNA inhibitors to miR-17-5p, miR-18a-5p, miR-29a-3p, miR-21-5p, and let-7a-5p. After the treatment for three passages, the downstream experiments such as H-NSCs senescence, proliferation, and differentiation were performed.

Western blot

H-NSCs whole protein was harvested by using RIPA lysis buffer supplemented with protease inhibitor cocktail (Roche) and Phosphatase inhibitor cocktail (Roche). H-NSCs nuclear protein and cytoplasmic protein were extracted by using the Nuclear and Cytoplasmic Protein Extraction Kit (Beyotime Biotechnology) following the manufacturer's protocols. The protocols for protein concentration mesurement and western blot were described above. The following antibodies were usde: p16^{INK4a} (1:1,000), rabbit monoclonal anti-P21 (1:1,000; Abcam, NO.ab109199), Rabbit polyclonal anti-P53 (1:1,000; Abcam, NO.ab131442), γ-H2AX (1:1,000), TFEB (1:1,000), LAMP1(1:500), rat monoclonal anti-LAMP2 (1:500; Santa Cruz, NO.sc-20004), rabbit monoclonal anti-S6K1(1:1,000; Abcam, NO.ab186753), rabbit anti-Phospho-p70 S6 Kinase (Thr389) (1:1,000; Cell Signaling Technology, NO.cst9205), rabbit monoclonal anti-β-actin (1:1,000; Abcam, NO.ab179467), mouse monoclonal anti-SYP (1:1,000; Abcam, NO.ab8049), mouse monoclonal anti-Psd-95 (1:1,000; Abcam, NO.ab13552), mouse monoclonal anti-Gap-43 (1:500; Santa Cruz, NO.sc-17790), mouse monoclonal anti-Syn-IIa (1:500; Santa Cruz, NO.sc-136086), mouse monoclonal anti-Histone H3 (1:1,000; Bioss, NO.bsm-33042M), mouse monoclonal anti-GAPDH (1:1,000; Abcam, NO.ab8245). The bands were imaged with a FluorChem M Fluorescent Imaging System and the gray value was analyzed by Image J software.

Statistical analysis

All data were presented as mean ± SEM. Student's t-test was employed to examine the inter-group differences, whereas one-way analysis of variance (ANOVA) were utilized

to explore the heterogeneity among different groups, followed by Bonferroni post hoc test in the absence of equivalent variance. A difference of $P < 0.05$ was deemed to be statistically significant.

Supplemental Table 1. Sequences of miRNA primers.

miRNA name	Product imformation	Mature miRNA Sequence (5'-3')
miR-17-5p	Hs_miR-17_2 miScript Primer Assay	CAAAGUGCUUACAGUGCAGGUAG
miR-18a-5p	Hs_miR-18a_2 miScript Primer Assay	UAAGGUGCAUCUAGUGCAGAUAG
miR-21-5p	Hs_miR-21_2 miScript Primer Assay	UAGCUUAUCAGACUGAUGUUGA
miR-29a-3p	Hs_miR-29a_1 miScript Primer Assay	UAGCACCAUCUGAAAUCGGUUA
let-7a-5p	Hs_let-7a_2 miScript Primer Assay	UGAGGUAGUAGGUUGUAUAGUU
RNU6-2	Hs_RNU6-2_11 miScript Primer Assay	ACGCAAATTCGTGAAGCGTT

Supplemental Table 2. Sequences of miRNA inhibitors.

miRNA inhibitor name	Sequence (5'-3')
miR-17-5p inhibitor	CUACCUGCACUGUAAGCACUUUG
miR-18a-5p inhibitor	CUAUCUGCACUAGAUGCACCUUA
miR-21-5p inhibitor	UCAACAUCAGUCUGAUAAGCUA
miR-29a-3p inhibitor	UAACCGAUUUCAGAUGGUGCUA
let-7a-5p inhibitor	AACUAUACAACCUACUACCUCA
Negative control	CAGUACUUUUGUGUAGUACAA

2. Results

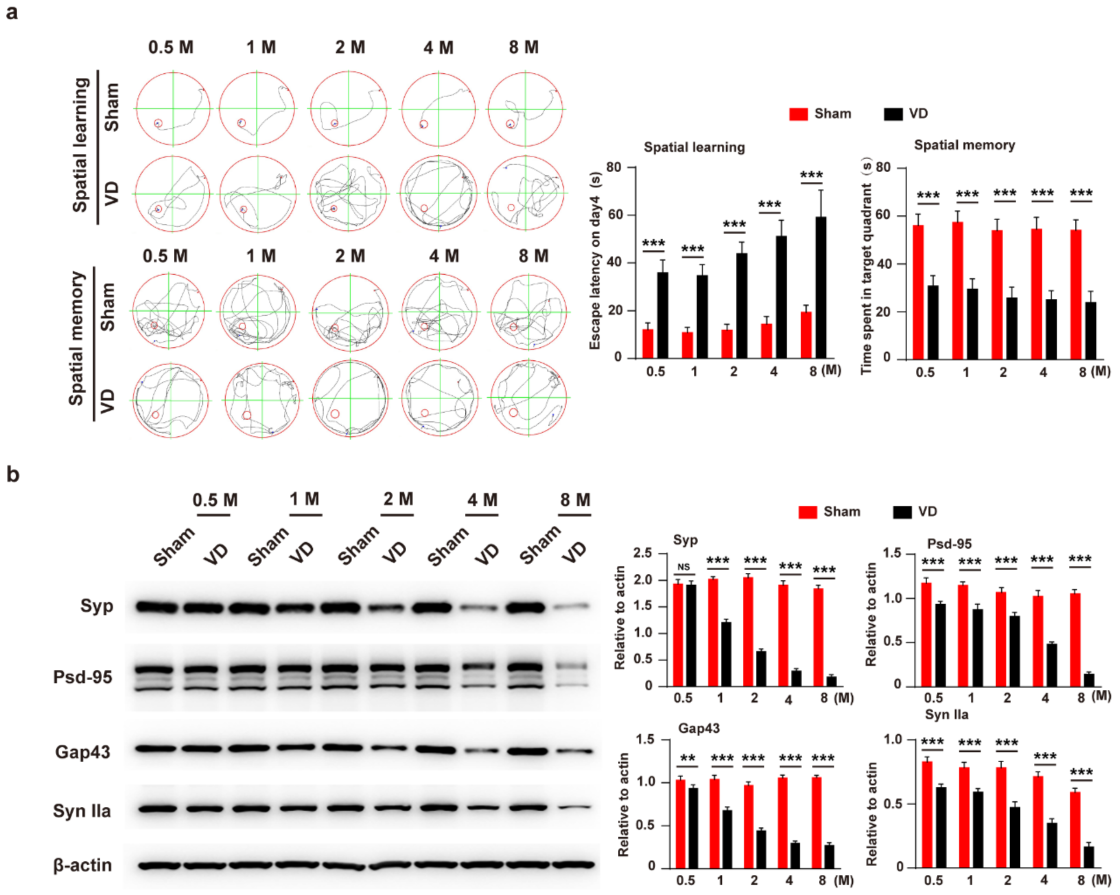

Supplemental Figure 1. Spatial learning and memory deficits as well as synaptic plasticity proteins reduction in VD. (a) Spatial learning and memory abilities in sham and VD group were tested by MWM. (n=10/group; ***P<0.001). (b) Western blot analysis and quantitative of Syp, GAP43, PSD95, and Syn IIa in hippocampus of sham and VD group at each time point. (n=6/group; **P<0.01, ***P<0.001).

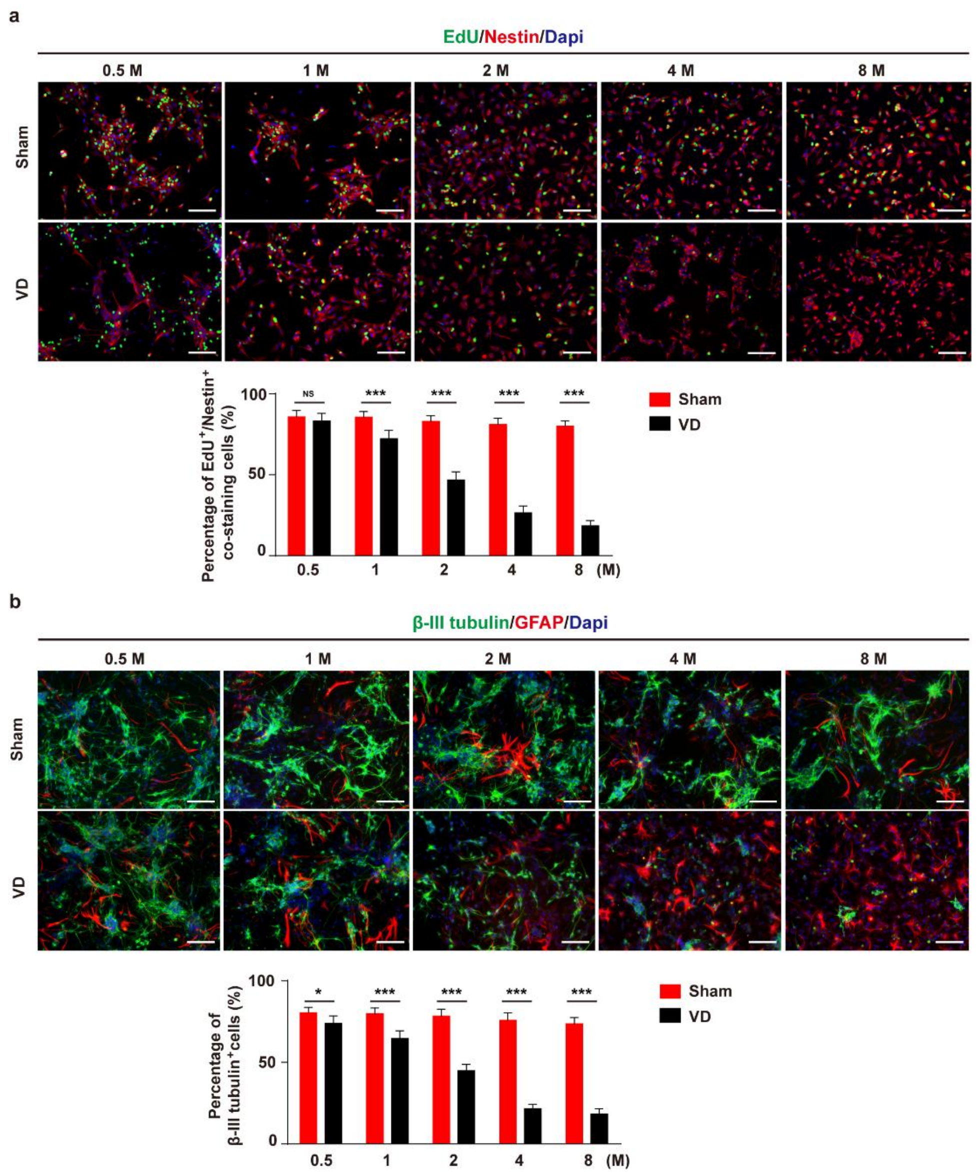

Supplemental Figure 2. H-NSCs proliferation and neuron differentiation deficits in VD. (a) Immunofluorescence staining of EdU incorporation and quantification of EdU$^+$/Nestin$^+$/Dapi$^+$ cells in Nestin$^+$/Dapi$^+$ cells in sham and VD group at each time point. (Scale bar, 100 μm; n=3/group; ***P<0.001). (b) Immunofluorescence staining of neuron differentiation in isolated H-NSCs and quantification of β-III tubulin$^+$ cells in whole cells in sham and VD group at each time point. (Scale bar, 100 μm; n=3/group; *P<0.05, ***P<0.001).

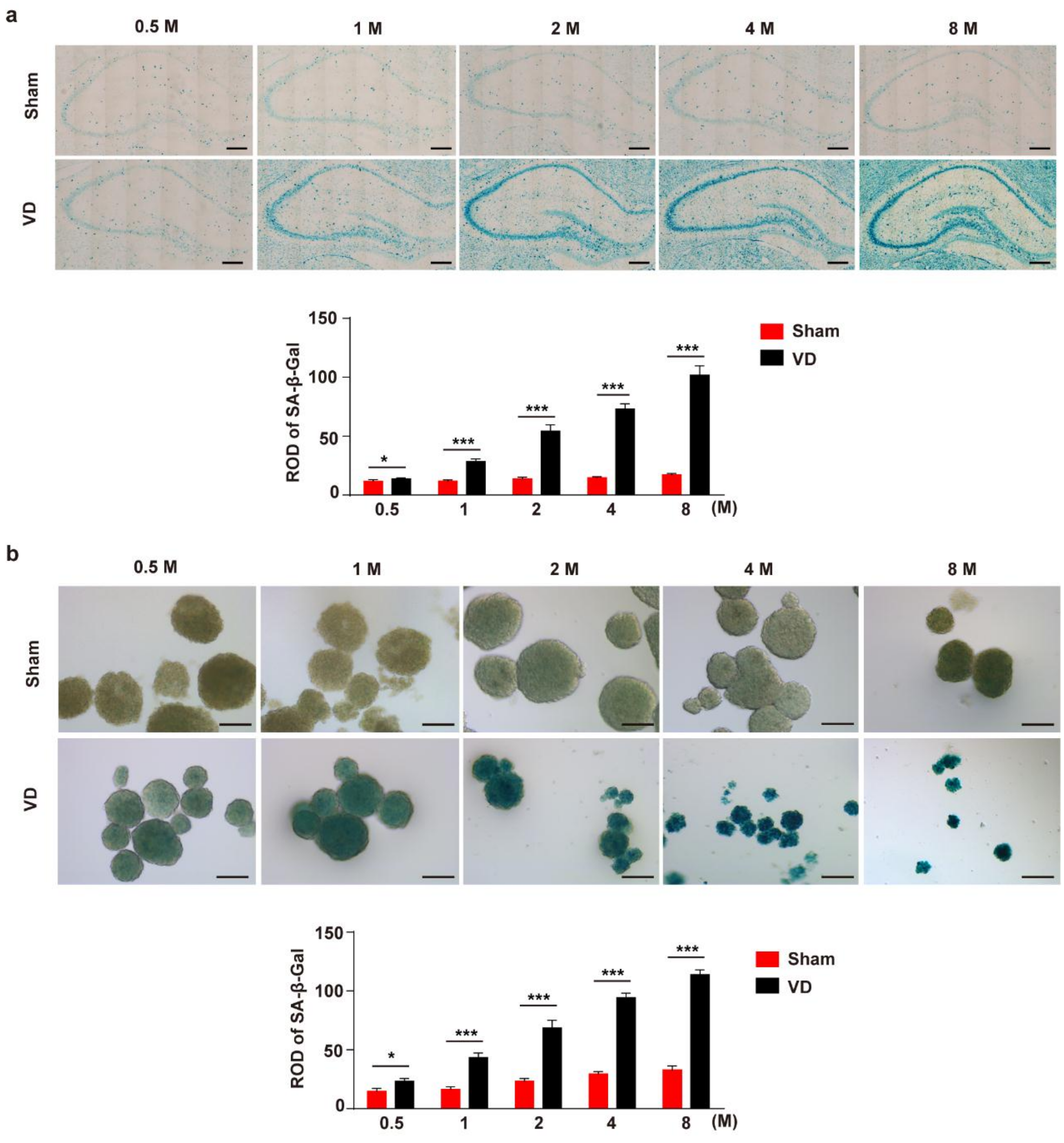

Supplemental Figure 3. Increasing of SA-β-gal staining in hippocampus and H-NSCs of VD rats. (a) SA-β-gal staining of hippocampus and quantification of SA-β-gal intensity in sham and VD group at each time point. (Scale bar, 400 μm; n=6/group; *P<0.05, ***P<0.001). (b) SA-β-gal staining of isolated neurospheres and quantification of SA-β-gal intensity in sham and VD group at each time point. (Scale bar, 100 μm; n=3/group; ***P<0.001).

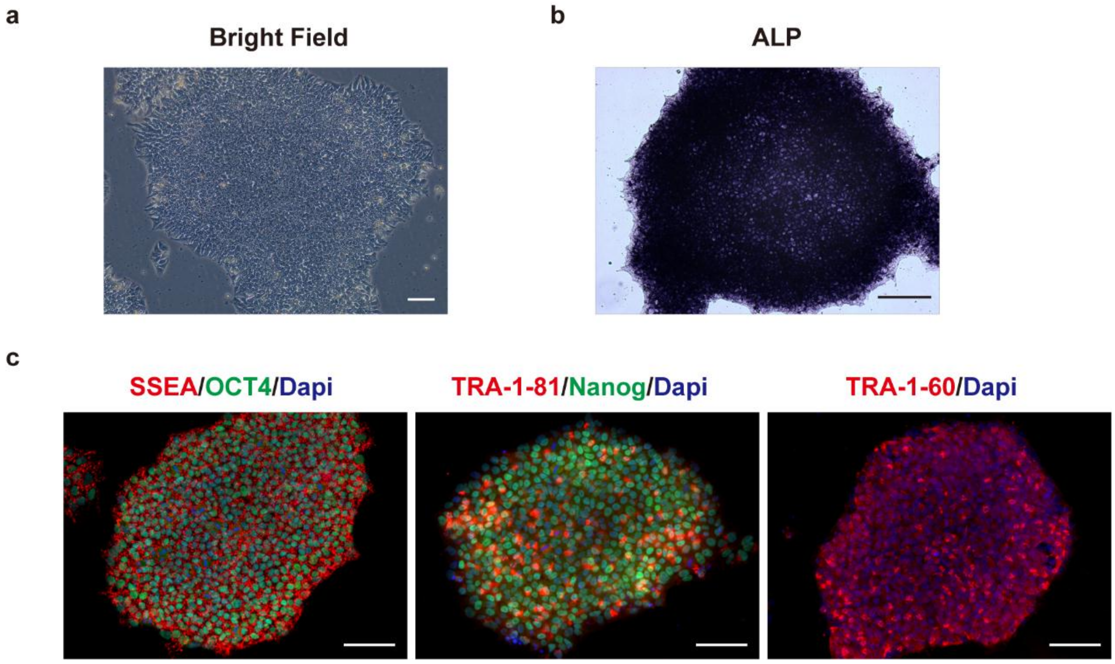

Supplemental Figure 4. Characterization of ESCs and ESC-sEVs. (a) ESCs colonies morphology in bright field. (Scale bar, 100 μm). (b) ALP staining of ESCs. (Scale bar, 150 μm). (c) Immunofluorescence staining of OCT4, Nanog, TRA-1-81, TRA-1-60, and SSEA4 in ESCs. (Scale bar, 100 μm).

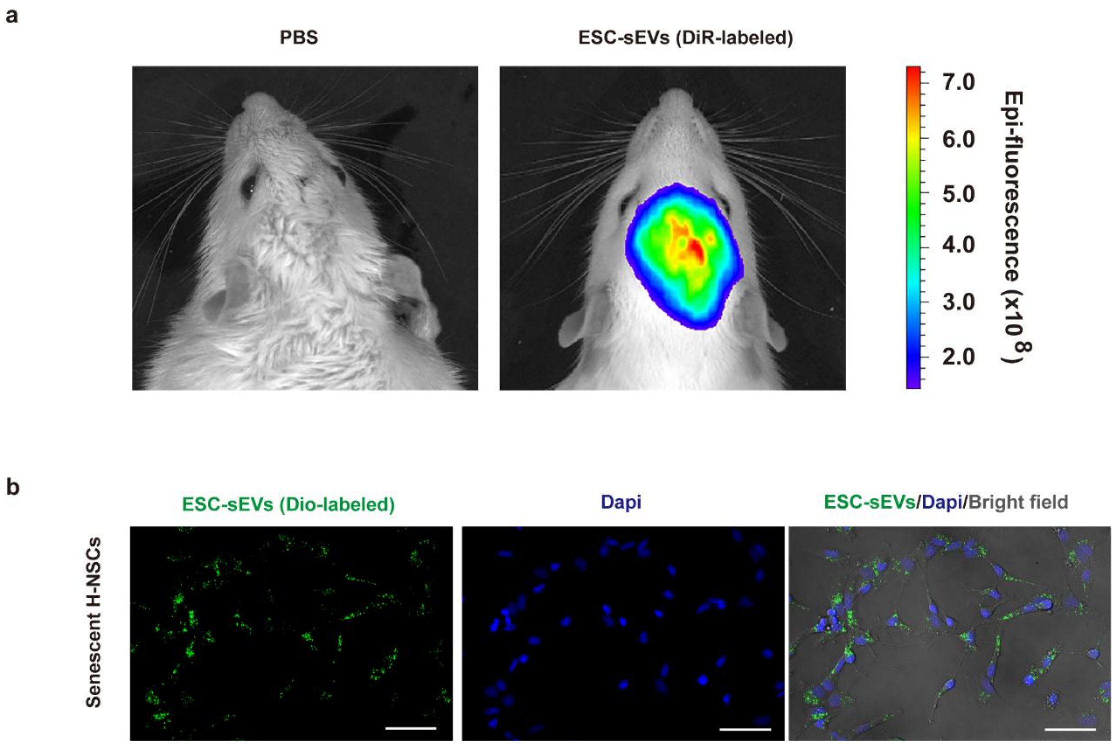

Supplemental Figure 5. (a) Representative fluorescent images of Rats' brains in PBS and DiR-ESC-sEVs group captured by IVIS Spectrum/CT imaging system. (n = 3). (b) Immunofluorescence staining of Dio-labeled ESC-sEVs (green) internalized by senescent H-NSCs (Scale bar, 50 μm; n = 3).

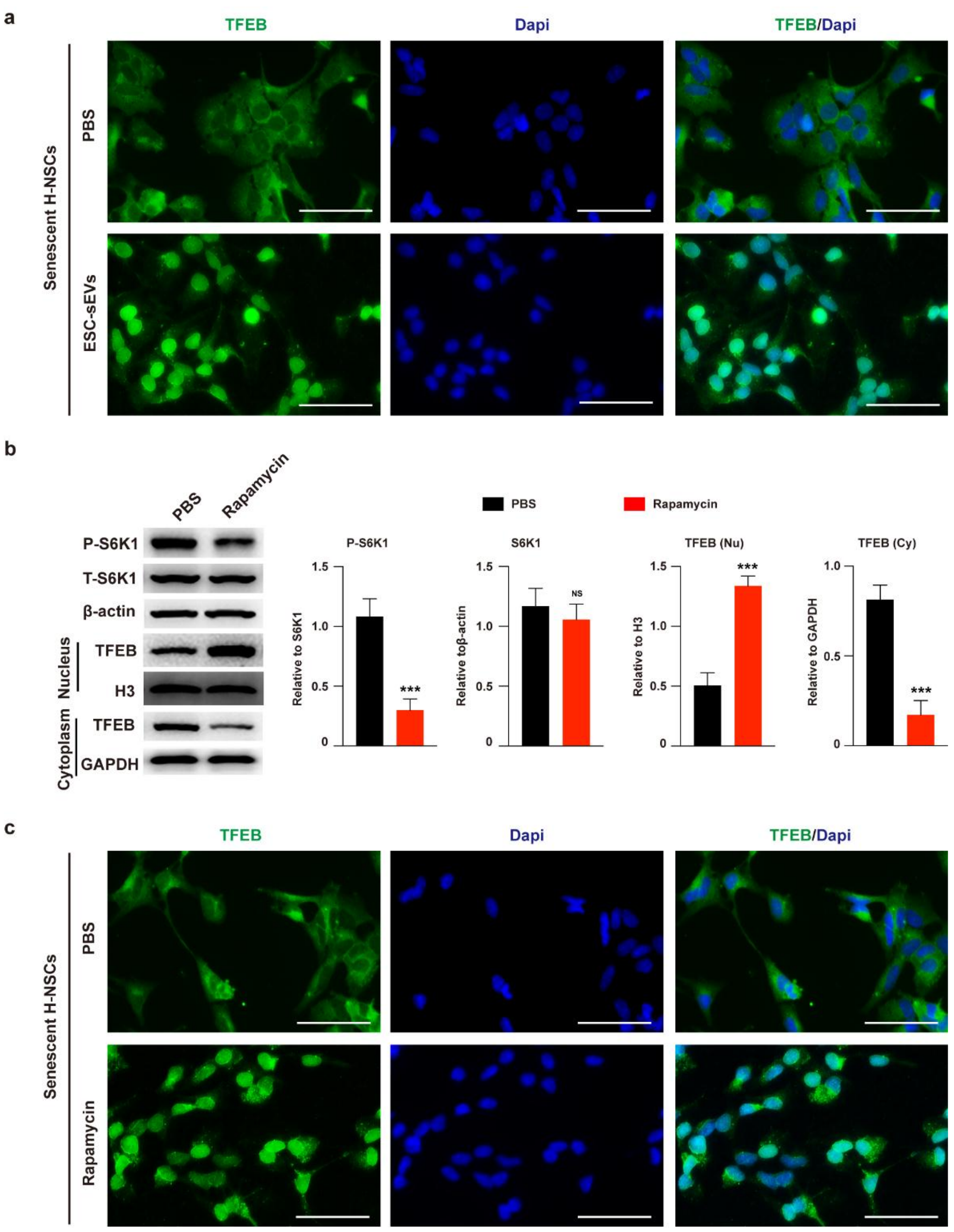

Supplemental Figure 6. (a)Immunofluorescence staining of TFEB location in ESC-sEVs or PBS treated senescent H-NSCs (Scale bar, 50 μm; n = 3). (b) Western blot analysis and quantification of P-S6K1, S6K1, nuclear TFEB, and cytoplasmic TFEB in senescent H-NSCs treated with rapamycin (25 nM) or PBS (n=3; ***P<0.001). (c) Immunofluorescence staining of TFEB location in rapamycin (25 nM) or PBS treated senescent H-NSCs (Scale bar, 50 μm; n = 3).

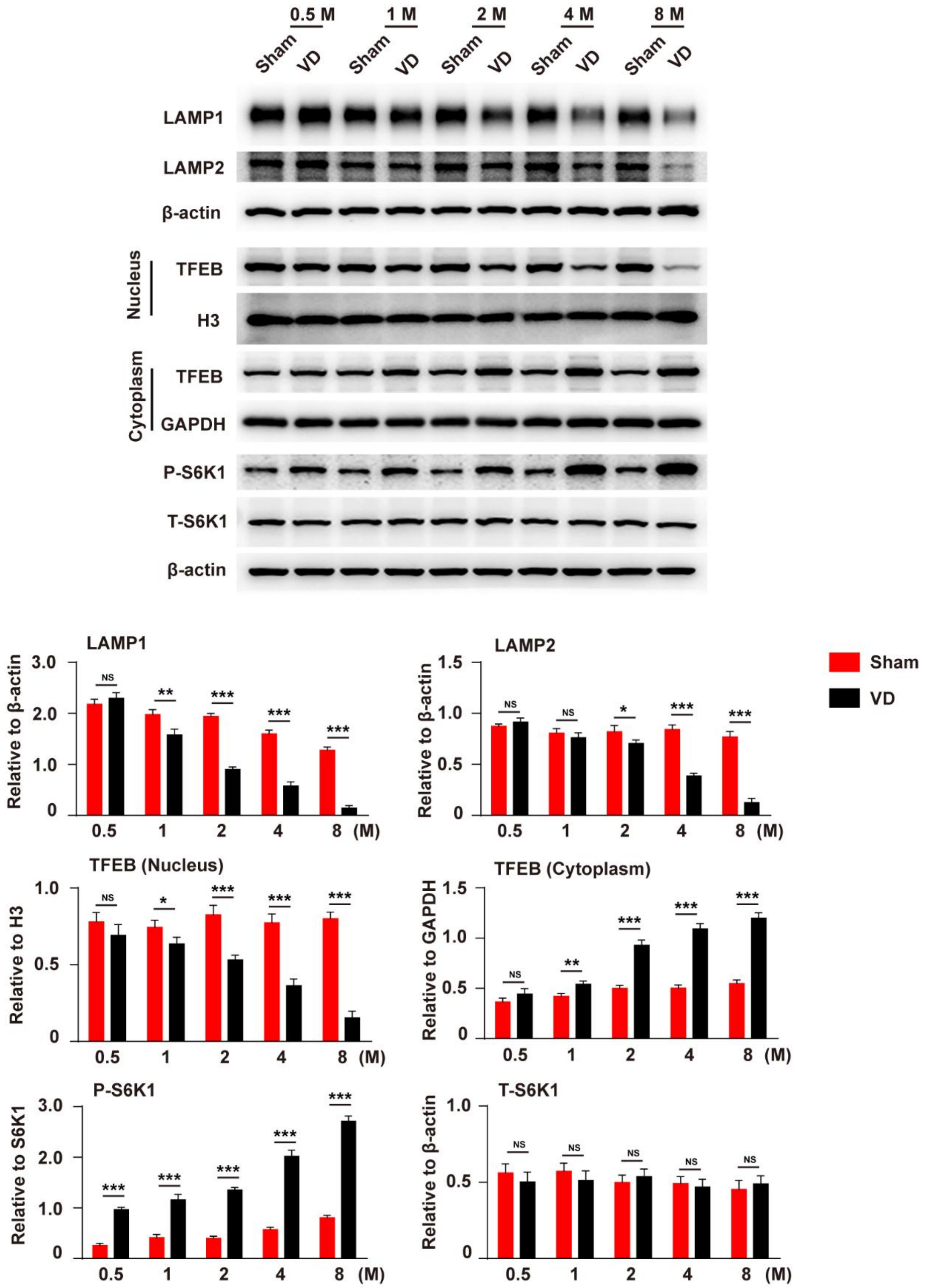

Supplemental Figure 7. Western blot analysis and quantification of LAMP1, LAMP2, nuclear TFEB, cytoplasmic TFEB, P-S6K1, and S6K1 in isolated H-NSCs in sham and VD group at each time point (n=3; *P<0.05, **P<0.01, ***P<0.001).

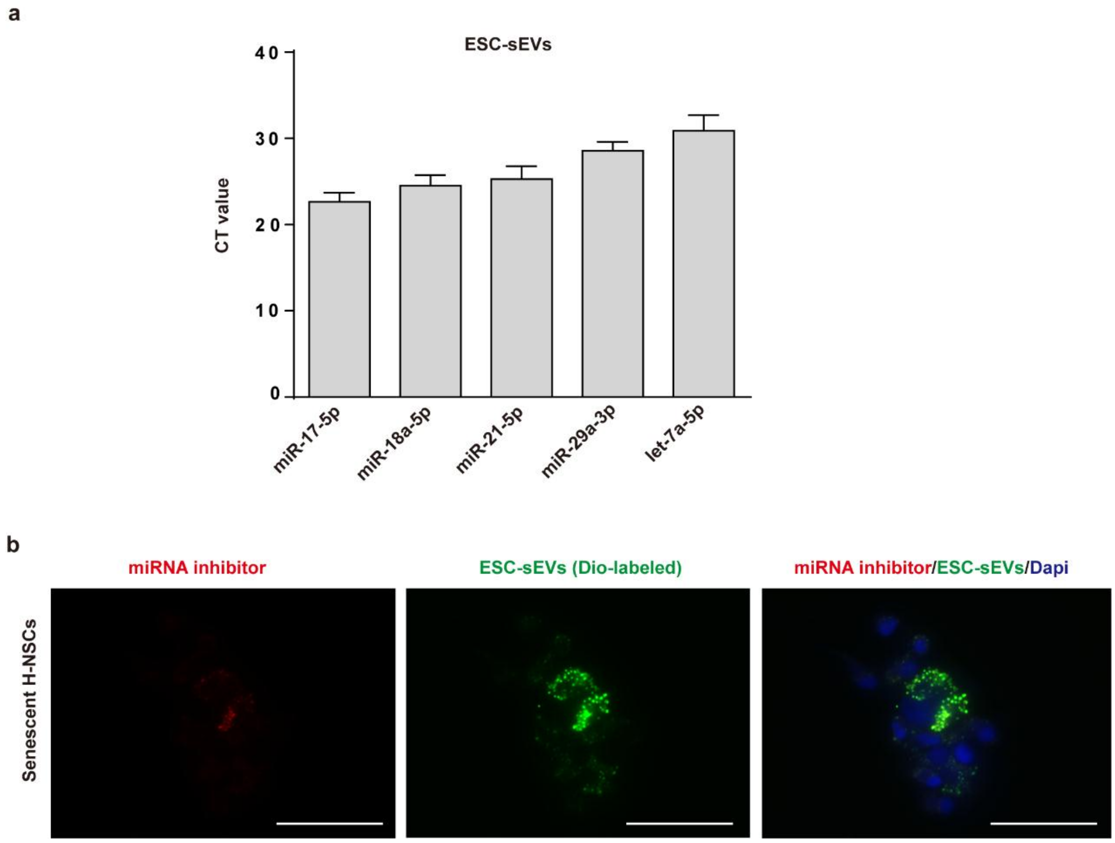

Supplemental Figure 8. (a) CT value of miR-17-5p, miR-18a-5p, miR-21-5p, miR-29a-3p, and let-7a-5p in ESC-sEVs by RT-qPCR analysis (n = 3). (b) Immunofluorescence staining of DIO-labeled ESC-sEVs (green) overlapped with Cy3-labeled miRNA inhibitors (red) internalized by senescent H-NSCs (Scale bar, 50 μm; n = 3).

References

[1] C. Thery, K.W. Witwer, E. Aikawa, M.J. Alcaraz, J.D. Anderson, R. Andriantsitohaina, A. Antoniou, T. Arab, F. Archer, G.K. Atkin-Smith, *et al*, *J Extracell Vesicles.* **2018**, 7,1535750.

[2] Y. Tian, L. Ma, M. Gong, G. Su, S. Zhu, W. Zhang, S. Wang, Z. Li, C. Chen, L. Li L, L. Wu, X. Yan, *ACS nano.* **2018**, 12, 671.

[3] N.S. Jiwa, P. Garrard, A.H. Hainsworth, *J Neurochem.* **2010**, 115, 814.

[4] N. Zhang, M. Xing, Y. Wang, H. Tao, Y. Cheng Y, *Neuroscience.* 2015, 311, 284.

[5] Q. Zhu, X. Ling, Y. Yang, J. Zhang, Q. Li, X. Niu, G. Hu, B. Chen, H. Li, Y. Wang, Z. Deng, *Adv Sci (Weinh).* **2019**, 6, 1801899.

[6] M. Costa-Mattioli, D. Gobert, H. Harding, B. Herdy, M. Azzi, M. Bruno,M. Bidinosti, C. Ben Mamou, E. Marcinkiewicz, M. Yoshida, H. Imataka, A.C. Cuello, N. Seidah, W. Sossin, J.C. Lacaille, D. Ron, K. Nader, N. Sonenberg, *Nature.* **2005**, 436, 1166.

[7] T. Kitayama, M. Yoneyama, K. Tamaki, Y. Yoneda, *J Neurosci Res.* 2004, 76, 599.

[8] B. Chen, Y. Sun, J. Zhang, Q. Zhu, Y. Yang, X. Niu, Z. Deng, Q. Li, Y. Wang, *Stem Cell Res Ther.* **2019**, 10, 142.

[9] L. Chen, H. Yao, X. Chen, Z. Wang, Y. Xiang, J. Xia, Y. Liu, Y. Wang, *Neurochem Res.* **2018**, 43, 430.
